# Review of the state of science and evaluation of currently available *in silico* prediction models for reproductive and developmental toxicity: A case study on pesticides

**DOI:** 10.1002/bdr2.2062

**Published:** 2022-06-24

**Authors:** Anastasia Weyrich, Madeleine Joel, Geertje Lewin, Thomas Hofmann, Markus Frericks

**Affiliations:** ^1^ Experimental Toxicology and Ecology BASF SE Ludwigshafen Germany; ^2^ Preclinical Science – Föll Mecklenburg & Partner GmbH Münster Germany; ^3^ Agricultural Solutions – Toxicology CP BASF SE Limburgerhof Germany

**Keywords:** *in silico* predictions, *in silico* protocols, pesticide, QSAR, reproductive toxicology

## Abstract

**Background:**

*In silico* methods for toxicity prediction have increased significantly in recent years due to the 3Rs principle. This also applies to predicting reproductive toxicology, which is one of the most critical factors in pesticide approval. The widely used quantitative structure–activity relationship (QSAR) models use experimental toxicity data to create a model that relates experimentally observed toxicity to molecular structures to predict toxicity. Aim of the study was to evaluate the available prediction models for developmental and reproductive toxicity regarding their strengths and weaknesses in a pesticide database.

**Methods:**

The reproductive toxicity of 315 pesticides, which have a GHS classification by ECHA, was compared with the prediction of different *in silico* models: VEGA, OECD (Q)SAR Toolbox, Leadscope Model Applier, and CASE Ultra by MultiCASE.

**Results:**

In all models, a large proportion (up to 77%) of all pesticides were outside the chemical space of the model. Analysis of the prediction of remaining pesticides revealed a balanced accuracy of the models between 0.48 and 0.66.

**Conclusion:**

Overall, predictions were only meaningful in rare cases and therefore always require evaluation by an expert. The critical factors were the underlying data and determination of molecular similarity, which offer great potential for improvement.

## 1 INTRODUCTION

Reproductive toxicity (Reprotoxicity) is one of the most critical factors in pesticide approval. Due to the 3R principle, the approval authorities are demanding more and more *in silico* evaluations for assessing reprotoxicity. Several models are available using generalized positive or negative calls not evaluating the particular endpoint or study design. This paper aims at discussing the difficulty and relevant parameters in designing adequate *in silico* models for developmental and reproductive toxicology. To illustrate the difficulties, available models have been tested using a database of 310 pesticides, which are data rich and where testing follows the OECD testing guidelines.

### 1.1 Complexity of reproductive toxicology

Reproductive toxicology (Reprotox) reflects the entire circle from formation and maturation of gametes through mating and conception, the embryonic and fetal development, postnatal adaptations, up to sexual maturation of the offspring. Due to the multitude of processes, pathophysiological disturbances may be observed as functional (e.g., altered estrous cyclicity, impaired reflex ontogeny) or structural (e.g., malformations, delayed bone ossification) anomalies or as behavioral alterations (e.g., missing mating drive, altered maternal behavior). Assessing all these factors in one study takes a very long time (approximately two years in rodents) and anomalies sometimes can hardly be appointed to a single interference. Therefore, the entire reproductive cycle is often broken down to several sections, each being tested separately. In this way, particular aspects can be assessed in more detail. However, the enormous animal consumption, time, and cost remain, and it is a great incentive for the development of alternative *in vitro* and *in silico* methods.

In the following section, the *in vivo* studies are presented based on their OECD guidelines, which must be carried out for the registration of a pesticide in the EU. Since these studies are a potential data basis for *in silico* models, knowledge of the assessed endpoints and their classification in the overall toxicological context is of great importance.

#### 1.1.1 Current OECD Guidelines for assessment of reprotoxicity

In the following section, the major study types used for the generation of reproductive and developmental toxicity data for pesticides and chemicals are outlined. The usual species used in these studies are rats as rodents and rabbits as non‐rodents. Additional study types like OECD 422, developmental neurotoxicity studies, or pharma study types are not covered but are equally important and contribute to the available database.

##### OECD 414: Prenatal Developmental Toxicity Study in one rodent and one non‐rodent (OECD, 2018a)

Young mature nulliparous rats are used. Animals in the estrous phase are mated overnight with a male. Successful mating is detected by sperms in the vaginal lavage and defines gestation day 0. Estrous phase in rabbits can be detected by reddening of the vulva if provoked by estrogen injection. Rabbits are mated with a male of proven fertility. Mating is confirmed by the presence of spermatozoa in the vaginal lavage. Alternatively, artificial insemination after hormone treatment can be performed in rabbits. Ovulation occurs approximately 10 hr after mating or estrogen injection.

Animals are then allocated to the different treatment groups (one control group, three treatment groups). Usually, each group consists of 22–24 animals to generate 20 litters per group. Treatment begins at implantation (Days 5–6 in rats and Day 6 in rabbits) and continues until the day before scheduled sacrifice. On Day 20/21 (rats) and 28/29 (rabbits), dependent on strain and/or laboratory, the animals are delivered by cesarean section. Cesarean section is done since otherwise malformed born pups would be lost by cannibalism.

During treatment, the behavior of the animals is carefully observed. Body weight and food consumption are recorded at regular intervals. At cesarean section, the uterus is opened. The numbers of corpora lutea, implantations, early and late resorptions as well as live and dead fetuses are determined. Individual fetal body weights are recorded. All fetuses are examined for external abnormalities. In rats, one half of the fetuses is examined for visceral alterations. The other fetuses are eviscerated, skinned, and evaluated for skeletal alterations, which is usually done by staining with Alcian Blue (cartilage) and Alizarin Red (bones). In rabbits, all fetuses are examined for visceral examination and then eviscerated, stained with Alcian Blue and Alizarin Red and examined for skeletal examination. External, visceral, and skeletal findings are usually classified as malformations (a permanent structural change that is likely to adversely affect the survival or health of the species under investigation) and variations (a change that occurs within the normal population under investigation and is unlikely to adversely affect survival or health).

There are two different study types available for assessing reprotoxicity, which are explained in the next section:

##### OECD 416: Two‐Generation Reproduction Toxicity Study in rodents (usually rats) (OECD, 2001)

The objective of this study is the determination of potential effects on maturation of gametes, mating, fertilization, pre‐implantation stages, implantation. Further potential adverse effects encompass estrous cycle, transport of the fertilized egg, pregnancy, birth, lactation, and growth of the offspring across two generations. In males, effects on libido and epididymal sperm maturation are possible, which cannot be detected otherwise.

Groups of 25 male and 25 virgin female rats, 5–9 weeks of age, are used in this study and allocated randomly to the treatment groups (one control group, three treatment groups). The animals are treated for 70 consecutive days (56 days in case of mice) prior to mating until sacrifice. This time covers a whole spermatogenic cycle including sperm maturation in the epididymis. After evidence of mating, that is, presence of spermatozoa in vaginal smears in the morning, the females are separated from the assigned male and allowed to deliver their F1 offspring. Standardization of offspring at postnatal day (PND) 4 is optional. After 3 weeks of lactation, the F1 animals are separated from their mothers, which are then euthanized. The uterus is opened, and the number of implantation scars is counted. Dosing of F1 animals is then continued for at least 10 weeks before they are mated. After evidence of mating, the females are separated and allowed to deliver their F2 offspring. Standardization of offspring at PND 4 is also optional here. After 3 weeks of lactation, the F2 animals and the maternal animals are euthanized. Males are euthanized when the mating outcome is sufficient. Reproductive organs are weighed and examined histopathologically.

Examined parameters consist of body weight, food consumption, estrous cycle determination, litter parameters, anogenital distance, developmental landmarks of F1 offspring (e.g., preputial separation, vaginal opening), and spermatological examinations. For this purpose, sperm samples are taken from the cauda epididymis and assessed for sperm concentration and sperm motility (motionless, locally motile and progressively motile). Alternatively, computer‐assisted sperm analysis (CASA) can be used. Additionally, testicular spermatid head count is determined after homogenization of the testis. Sperm morphology is evaluated by assessment of abnormal head, mid‐piece, and tail.

Recently, EFSA required the assessment of nipple retention in male pups around PND 14.

##### OECD 443: Extended One‐Generation Reproductive Toxicity Study in rodents (usually rats) (OECD, 2018b)

This study design originally has been discussed as a replacement for the Two‐Generation Reproduction Toxicity Study design, as it requires considerably less animals. The study design is similar to the Two‐Generation Reproduction Toxicity Study, but ideally covers only the F1 generation. Optionally developmental neurotoxicity cohorts and a developmental immunotoxicity can be added in the F1 generation.

Groups of 25 male and 25 virgin female rats are used in this study and allocated randomly to the different treatment groups. Pre‐mating treatment is at least 2 weeks in males and females. In practice, sometimes a 10‐week pre‐mating treatment is required by authorities. The age of the animals depends on the pre‐mating period (10 weeks treatment: 5–6 weeks old; 2 weeks treatment: 11–12 weeks old). After evidence of mating, the females are separated from the assigned male and allowed to deliver their F1 offspring. Anogenital distance in both sexes and nipple retention are assessed in males. Standardization of offspring at PND 4 is optional. After total 10 weeks of treatment, hematological and clinical chemistry examinations, urinalysis, assessment of organ weights, and histological examination of numerous organs are carried out in the parental animals. Spermatological examinations as described for the OECD 416 study are performed. At weaning, offspring are assigned to the following cohorts:

F1‐1A (Reprotoxicity): These animals (20 females and 20 males) are dosed daily from PND 22 and euthanized at the age of 13 weeks and examined in the same way as the parental generation.
F1‐1B (Reprotoxicity): These animals (20 females and 20 males) are dosed daily from PND 22 and euthanized at the age of 14 weeks. Reproductive organs and a limited number of other organs are weighed and preserved for possible histopathological examination. If there is evidence of a change of reproductive parameters in the F1A cohort, which warrants further data, these animals are used for breeding and generation of a F2 generation, which is raised and examined like the F1 offspring. It should be noted (although not mentioned in the guideline) that in this case 20 F2 litters should be produced. Therefore, it may be prudent to increase the size of the F2 generation to 25 males and 25 females and consequently increase also the number of parental animals.
F1‐2A (optional) Developmental Neurotoxicity: These animals (10 males and 10 females; one male or 1 female out of 20 litters) are subjected to detailed neurological examinations (functional observation battery, motor activity). They are euthanized in Weeks 11 and 12 by perfusion fixation. Central and parts of the peripheral nervous system are preserved, fixed, and embedded in paraplat or plastic (epoxy resin) and histologically examined.
F1‐2B (optional) Developmental neurotoxicity: These animals (10 males and 10 females; one male or 1 female out of 20 litters) are euthanized on PND 22, undergo perfusion fixation and are used for assessment of brain weight and histological examination of brain and brain‐associated structures.
F1‐3 (optional) Developmental immunotoxicity: These animals (10 males and 10 females; one male or 1 female out of 20 litters) are used at PND 56 ± 3 in a T‐cell‐dependent antibody response assay (TDAR), for example, the primary IgM antibody response to a T‐cell‐dependent antigen, such as Sheep Red Blood Cells or Keyhole Limpet Hemocyanin. Additional pups may be required from the control group to act as positive control animals in TDAR. The response is evaluated by counting specific plaque‐forming cells in the spleen or by determining the titer of SRBC‐ or KLH‐specific IgM antibody in the serum by ELISA, at the peak of the response.

##### Alternative study designs

Many alternative *in vivo* non‐mammalian and *in vitro* approaches to contribute to the 3Rs concept (Russell & Burch, 1959) exist, but none is accepted by regulatory agencies as alternative test system for registration of pesticides. The major drawback of these alternatives is that the interaction with the maternal compartment is missing.

However, since these methods are of great interest in current research, examples are mentioned for the sake of completeness:

- the Zebra fish embryotoxicity test (ZET) (Selderslaghs, Van Rompay, De Coen, & Witters, 2009)
- the frog embryo teratogenesis assay Xenopus (FETAX) (Bantle, Fort, & James, 1989)
- the whole embryo culture test (WEC) (Piersma et al., 2004)
- the embryonic stem cell test (EST) (Seiler & Spielmann, 2011)

#### 1.1.2 Differences between study guidelines

One aspect that is often not considered in the comparison of toxicity studies is the change in the underlying experimental guidelines. In case of reprotoxicity, this can have tremendous impact. For example, the original versions of the OECD 414: Developmental Toxicity Testing guideline required dosing only during embryogenesis and organogenesis. In the rat, this is between gestation days (GD) 6–15; in the rabbit 6–19. In the more recent guideline, this was adapted to also cover the later intrauterine maturation leading to treatment between GD 6–20 in the rat and 6–29 in the rabbit. In both guideline versions, the animals were euthanized and delivered by cesarian section to achieve a standardized read out. By use of the older study design developmental delays, for example, ossification effects might have recovered by the last day of pregnancy. This “recovery” period is not present in the newer test design. Furthermore, the day of cesarean section varies between GD 20 and 21 in rats and GD 29 and 30 in rabbits among laboratories and between animal strains. Especially in rats, this difference has significant impact on the ossification status and fetal weight. Since dosing is based on the dam's weight and fetal weight becomes a significant part of this in the last stage of gestation, the high dose tolerated by the dams is expected to be lower in the new study design in many cases.

Another change in the guideline with strong impact is that in older studies, often only bone was stained. In more recent experiments, a co‐staining for cartilage is often applied, which allows a much better, standardized analysis of ossification effects.

The impact of guideline changes is even more prominent in OECD 416 (2‐generation study). A significant array of additional parameters has been added. Many of these are related to sexual maturation and endocrine disruption, such as anogenital distance, nipple retention, vaginal opening, and preputial separation. More recent changes include measurement of thyroid hormones. Therefore, results for these endpoints are not available for historic studies performed according to the old OECD protocols. In the meantime, depending on the regulatory framework, the OECD 416 is often replaced by the OECD 443. The assessed reprotoxic endpoints in both studies are similar, but in the OECD 443 the F2 generation is often avoided unless triggers are calling for generation of the F2. Additionally, the OECD 443 can contain cohorts for the assessment of developmental neurotoxicity and immunotoxicity.

Additional critical parameters are dose setting, which in historic times often used large spacings, for example, 100, 500, and 5,000 ppm. If the top dose showed excessive toxicity, and the low dose displayed no effects, the extend of effects on reproductive performance or sexual maturation cannot be clearly defined. For NOAEL setting this is not a problem, but for hazard and risk assessment, and also for QSAR, the widely spaced dose setting can mask effects at lower toxicity levels.

In addition to the OECD guidelines just presented, which are used for the classification of pesticides and chemicals in the EU, there are further guidelines for assessing reprotoxicity:

- US EPA OPPTS for the risk assessment of chemicals and pesticides
- ICH‐Guidelines for risk assessment for drug authorization, used in the EU (EMA), US (FDA), and Japan (MHLW)

These guidelines display great similarity in their general structure, but there are also some differences about the exposure period and the points considered.

#### 1.1.3 Causes and mechanisms of reprotoxicity

##### ADME during gestation and lactation

Critical points in reprotox that have not been adequately explored are ADME and metabolism. The fetuses and pups have different exposure conditions (De Schaepdrijver, Annaert, & Chen, 2019). In utero, exposure to the fetus is largely mediated by maternal supply via the placenta (Tetro, Moushaev, Rubinchik‐Stern, & Eyal, 2018). The exposure will therefore be restricted to bioavailable active ingredients and their bioavailable metabolites. Usually, metabolism information is only available for non‐pregnant animals. Due to the physiological changes in pregnancy, ADME parameters between pregnant and non‐pregnant animals can be significantly different, which can lead to an unknown pattern of exposure (Avram, 2020; Tasnif, Morado, & Hebert, 2016). In order to bundle knowledge about the metabolism of pesticides, EFSA initiated the creation of MetaPath with the EU transparency regulation, which can be used in the future, among other things, for the development of PBPK models.

Placental transfer can be a limiting factor for distribution, since the placenta is designed to form a protective barrier protecting the fetus from xenobiotic compounds. A number of models for placental transport have been proposed and can potentially contribute to an assessment of the fetal exposure situation.

The exposure of the offspring is initially via the meconium, maternal skin contacts and if the compound is fat soluble via milk. Only after pups start ingesting food, approximately around Days 10–14, does dietary exposure become a dominant factor. Currently, no systematic database for milk transfer is available across pharmaceuticals, pesticides, and chemicals. Therefore, log*p* values are a logical way of approximation. Here again, the metabolism of parent and data on tissue distribution into fat should be taken into account, which has not been systematically collected. Having a respective database would be a valuable addition into the toolset of PBPK models to evaluate.

An additional important parameter is the difference in the expression and activity of phase I—III enzymes. Fetal and pup metabolism and excretion is often limited while immature. For example, most transporters only reach maximal expression at around PND 21 in both liver and kidney. While significant data on the rat are available for a number of phase I—III enzymes, the database for humans and rabbits, as the second relevant species for teratogenicity testing, is limited (De Schaepdrijver et al., 2019).

All in all, too little is known about the exact ADME of pesticides during gestation or lactation. Whether the pesticides cross the placental barrier are metabolized by the fetus, exposure takes place via the milk or how ADME works in the pup are questions that cannot be answered for most pesticides. To make matters worse, the embryo/fetus/pup changes over the entire period under consideration, which is why it can be assumed that this also applies to ADME.

##### Importance of maternal toxicity and species differences

Based on the complexity of reprotoxicity, a vast interplay with related areas such as pathology, endocrinology, and general toxicology is necessary. To make the matter even more complex, an interrelation between generation effects can be seen, such as impaired maternal nutritional status leading to lower numbers of follicles maturing, lower reproductive success, and subsequent lower numbers of live born pups (Khera, 1987; Nitzsche, 2017; Theunissen et al., 2016). Or maternal toxicity can lead to a less than optimal uterine environment, lower nutritional supply to the fetus, possibly resulting in lower fetal weight and delayed skeletal ossification. The inherent role of maternal toxicity has gained increasing attention in the last decade as it assists data interpretation.

Furthermore, different animal models respond differently to exogenous stress factors. While rabbits for example often react with abortions, rats tend to maintain their pregnancies but may display higher numbers of resorptions, lower fetal weight, and developmental delay in their offspring. An additional factor, which is often overlooked, is the documentation of negative results, parameter that was assessed but is not affected. In the future, not only the documentation but also the publication of those can help to shed light on affected pathways.

Therefore, for each reprotoxicity assessment, the right time frame and route of exposure, the most appropriate animal model and a well‐suited laboratory with sufficient experience have to be carefully selected.

##### Adverse outcome pathways

While the conservative study approach can connect between an exposure at a certain timepoint and an outcome, it usually gives no clear information on the mode of action of adverse outcomes. For this, a different approach was developed.

In order to sort parameters, connect cause and consequences and subsequently organize scientific knowledge, the conceptual framework of Adverse Outcome Pathways (AOPs) was initiated. They are intended to aggregate knowledge currently dispersed in various sources from case studies, journal articles to databases into a systematic and accessible format that facilitates use of that knowledge.

AOPs are based of several principles:

- Linking a molecular initiating event (MIE) via several key events to an adverse outcome.
- Modular AOPs can assemble into AOP networks.
- And AOPs are living documents, reflecting the current state of science and open to evolutions as knowledge increases.

In addition to evidence supporting a causal relationship between different events, authors are also encouraged to provide quantitative understanding of the linkage, based upon 1. Response–response relationships, time scales, known modulating factors and known positive or negative feedback loops (Society for the Advancement of Adverse Outcome Pathways, 2022).

Adverse Outcome Pathways have gained increasing regulatory acceptance but still the number of OECD approved AOPs in reprotox is low and currently limited to:

- Androgen receptor agonism leading to reproductive dysfunction
- Aromatase inhibition leading to reproductive dysfunction
- Aryl hydrocarbon receptor activation leading to early life stage mortality, via increased COX‐2 or VEGF
- Inhibition of thyroperoxidase and subsequent adverse neurodevelopmental outcomes
- Histone deacetylase inhibition leading to testicular atrophy

Several additional molecular initiating events and their pathways are currently under review or open for adoption, such as histone deacetylase inhibition, estrogen receptor antagonism and PPARα activation. Nevertheless, major pathophysiological pathways for which teratogenic properties are known, still lack incorporation into the various adverse outcome networks, these include but are not limited to fetal anemia, HDAC (histone deacetylase) inhibition or methemoglobinemia, all affecting tissue differentiation.

### 1.2 *In silico* models

In the directive 2010/63/EU, the European Parliament defined the Three Rs principle, described first by Russell & Burch, 1959, as aim for the protection of animals used for scientific purposes in EU. To fulfill this aim, the member states should support the research on alternative methods. At that timepoint, the focus was mainly on *in vitro* methods but with the inure of REACH regulation in 2007 the *in silico* methods became more important due to the huge amount of additional animal tests requested. For this purpose, five OECD principles were published, which have to be fulfilled by regulatory used QSARs: (a) defined endpoints; (b) unambiguous algorithm; (c) defined domain of applicability; (d) appropriate measures of goodness‐of‐fit, robustness, and predictivity; and (e) a mechanistic interpretation (OECD, 2006).

The great advantages of *in silico* methods are the reduction of test animals and costs and high throughput compared to animal studies (Valerio, 2009). Hence, these methods are suitable for compound selection in early developmental steps or to fill existing gaps in empirical data. This makes *in silico* methods particularly attractive for reprotox, even if the prediction is difficult due to the number and complexity of the endpoints (Hewitt, Ellison, Enoch, Madden, & Cronin, 2010). The big challenges for *in silico* prediction of reprotoxicity endpoints are the complexity of ontogenesis, the combination of several endpoints with partly unknown AOPs and the limited availability of empirical reprotoxicity data (Cronin & Worth, 2008).

#### 1.2.1 Model types

The available *in silico* models for reprotoxicity endpoints, which were tested in this study, is mainly Structural Alerts (SAs) and rule‐based models or Quantitative Structure–Activity Relationship (QSAR) models.

SAs are chemical structures, which have been linked to toxic events (Yang, Lou, Li, Liu, & Tang, 2020). These alerts could be based on human expert knowledge (rule‐based models) or generated by machine learning (Venkatapathy & Wang, 2013). Also, mixtures of both methods are common. The advantages of these models are that they are easy to interpret and allow to localize the crucial structure for toxicity. Limitations are that the methods just show the presence or absence of SAs, and absent SAs are always interpreted as non‐toxicant even when based solely on incompleteness of SA lists. Besides, biological pathways of toxicity are not considered (Raies & Bajic, 2016).

QSAR models are based on the assumption that molecules that have a similar chemical structure tend to produce similar toxic effects (Hansch & Fujita, 1964). The description of the chemical structure and assessment of the similarity therefore play a decisive role in the creation of statistical models, which are created with a training data set of sample molecules with known toxicity (Valerio, 2009). The molecules can be described by molecular descriptors, which are based on the geometric, electronic, topological, constitutional, and thermodynamic properties of the molecule (Danishuddin & Khan, 2016). However, 2D fingerprints are often used to describe the chemical structure in the form of a bit vector. In the substructure keys‐based fingerprints, each bit represents the presence or absence of a predefined substructure (Cereto‐Massagué et al., 2015). In contrast, topological or path‐based fingerprints work by analyzing all fragments of the molecule following a path up to a certain number of bonds and then hashing each of those paths to create the fingerprint. Circular fingerprints are also hashed topological fingerprints, but they do not describe the path but the area around each atom up to a certain radius (examples for each type of fingerprint with description can be found in Table S5). Since the descriptors and the various molecular fingerprints differ greatly in their description of the molecules, this has a great influence on the functionality of the QSAR model. In order to combine the advantages of the various methods, combinations of several descriptors and a fingerprint are often used for building a QSAR model.

An alternative approach to define similarity is the use of compound class specific substructures or toxicophores (SMARTS), which can be combined with structural alerts or fingerprint techniques. This is a particular powerful approach for read across as it captures compound class intrinsic information (Enoch et al., 2022).

Significant efforts have also been invested to use bioactivity data, such as Toxcast or PubChem bioactivity data as an alternative type of descriptor. Such an affinity fingerprint is the vector consisting of compounds affinity or potency against a reference panel of proteins targets (Škuta et al., 2020). In a similar approach also effects from subchronic or chronic studies can be used. These approaches however are generally limited to marketed compounds or face the problem that bioactivity databases are proprietary information, for example, from pharmaceutical companies.

The characteristics of the models used in the study are briefly described in Table 1. A detailed description is given in Section 2.2.

**TABLE 1:** Short description of all tested models for the prediction of reprotoxicity

Platform	Model	Functional principle	Database	References
VEGA	Developmental Toxicity model (CAESAR, v.2.1.7)	QSAR classification model 13 EPA descriptors were used to describe molecular properties Classifier: Random Forest	292 chemical compounds (mainly drug data) 201 developmental toxicants/91 nondevelopmental toxicants Developmental toxicity was defined by FDA categories: A or B ➔ non‐toxicant C, D or X ➔ toxicant	Cassano & Benfenati (2010), Cassano et al. (2010)
	Developmental/Reproductive Toxicity library (PG, v.1.1.0)	Empirically based decision tree Expert rule based structural features Molecules could be classified into 25 different categories with known DART No categories for nontoxic chemicals Detailed description of the categories could be found in appendix II of Wu et al. (2013)	Decision tree is based on a data set of 716 chemicals (664 toxic, 16 non‐toxic) Detailed information about the chemicals and the references, on the basis of which they were classified, could be found in appendix I of Wu et al. (2013)	Benfenati (2020), Wu et al. (2013)
OECD (Q)SAR Toolbox	Expert‐based DART scheme	Is also based on the categories from Wu et al. (2013) like the PG model Further development of the categories	Same data base as PG model	OECD (Q)SAR Toolbox (2020), Wu et al. (2013)
Leadscope model applier	Repro Female Rat (RFR) v2	Statistical based model (QSAR) Three QSAR models were built with a balance of positive and negative compounds ➔ prediction is the average of all three model results Negative and positive features were identified The predicted positive probability is based on individual contributions from the model features Threshold in predicted positive probability is used to assign a positive or negative prediction	Includes adverse effects to female reproductive organs (cervix, fallopian tube, ovary, uterus, and vagina) and fertility Based on ICSAS database described by Matthews, Kruhlak, Cimino, Benz, and Contrera (2006a) 894 training compounds (14.8% positives)	Leadscope (2021), Matthews et al. (2006a), Matthews, Kruhlak, Cimino, Benz, and Contrera (2006b)
	Repro Male Rat (RMR) v2		Includes adverse effects to male reproductive organs (Cowper's gland, epididymis, prostate, seminal vesicles, and testes) and fertility Based on ICSAS database described by Matthews et al. (2006a) 714 training compounds (30.07% positives)	
CASE Ultra	Foetal Dysmorphogenesis (FDYSM) Rabbit	Statistical based SA model Local QSAR for each alert with physicochemical descriptors Outcome of a SAR prediction is given as the probability of being reprotoxic on a scale of 0 to 1 Specific classification threshold for each model Activating and deactivating alerts were detected	Pre‐processing of data and endpoints are described by Matthews et al. (2006a) 128 active/129 inactive	Chakravarti, Saiakhov, and Klopman (2012), Cioffi (2019a, 2019b, 2019c, 2019d), Matthews et al. (2006a), Matthews, Kruhlak, Daniel Benz, and Contrera (2007), Matthews, Kruhlak, Daniel Benz, Ivanov, et al. (2007)
	FDYSM Rat		436 active/457 inactive	
	Female fertility (FFERT) Rat		113 active/113 inactive	
	Male fertility (MFERT) Rat		180 active/180 inactive	

#### 1.2.2 Importance of molecular similarity

When using prediction models, the determination of molecular similarity is of enormous importance. On one hand, this is used in QSAR models to predict toxicity and, on the other hand, it can be used for all model types to determine the applicability domain (AD). The AD is the structure space on which the training set of the model was developed, to which it is applicable to make predictions for new compounds and therefore a good benchmark if the prediction is reliable.

The choice of the description of the molecule thus has a major influence on both the prediction and its evaluation. Mellor et al. showed that the notion of fingerprint‐derived similarity varies widely between data sets and structure types (Mellor et al., 2019). In particular, the subtle differences between very similar structures can often be overlooked, resulting in the same numerical similarity for such compounds. The descriptors and fingerprints for a model should therefore be selected with great care and the prediction of existing models should be critically examined by experts.

#### 1.2.3 Problems of prediction models for reprotoxicity

Especially for the prediction of reprotoxicity, the currently available *in silico* models have some weaknesses (Cronin & Worth, 2008). These are discussed in the following list:

- Many models only differentiate between toxic for reproduction or not. Since there can be many different MOAs with different conspicuous endpoints behind reprotoxicity, this information is very simplified. The use of models that only refer to *individual endpoints or parts of reprotoxicity* (e.g., female fertility) is therefore more promising.
- The current prediction models only consider *ADME* of pesticides indirectly via models trained on *in vivo* data. However, this is insufficient, considering, for example, the changes in the guidelines regarding exposure patterns, which can have an impact on ADME. There are currently no reliable models that can predict ADME during gestation. However, since this can greatly change the toxicity, ADME should at best be included in the models.
- To create a meaningful QSAR model, a *good quality database* is required, which should also be as comprehensive as possible. There is a lack of such data for reprotoxicity, especially since the type of data and their interpretation has changed significantly over the decades, for example, the changes in the guidelines (endpoints and dosage) and the interpretation of maternal toxicity.
- *Chemical similarity* is usually used to create QSAR models. Alternatively, or additionally, information about the compound class, biological activity or SMARTS, for example, could also be used. For example, the HPPD inhibitor group of herbicides has a significant structural heterogenicity, but all are increasing tyrosine in the rat leading to respective tyrosine mediated toxicity.
- For the creation of predictive expert based structural alert models, knowledge about the *AOPs* is crucial in order to be able to correctly name the relevant structural features. Since there is still a knowledge gap for reprotox and only four AOPs have so far been recognized by the regulatory authorities, these models tend to lead to incorrect predictions.

### 1.3 Testing the performance of prediction models for reprotoxicity

As discussed in the previous section, there are several challenges in predicting reprotoxicity using *in silico* models. Nevertheless, there are some commercial or freely available prediction models, which are tested in the following case studies with regard to their performance in predicting pesticides.

## 2 MATERIALS AND METHODS

### 2.1 Pesticide data base

**FIGURE 1:**
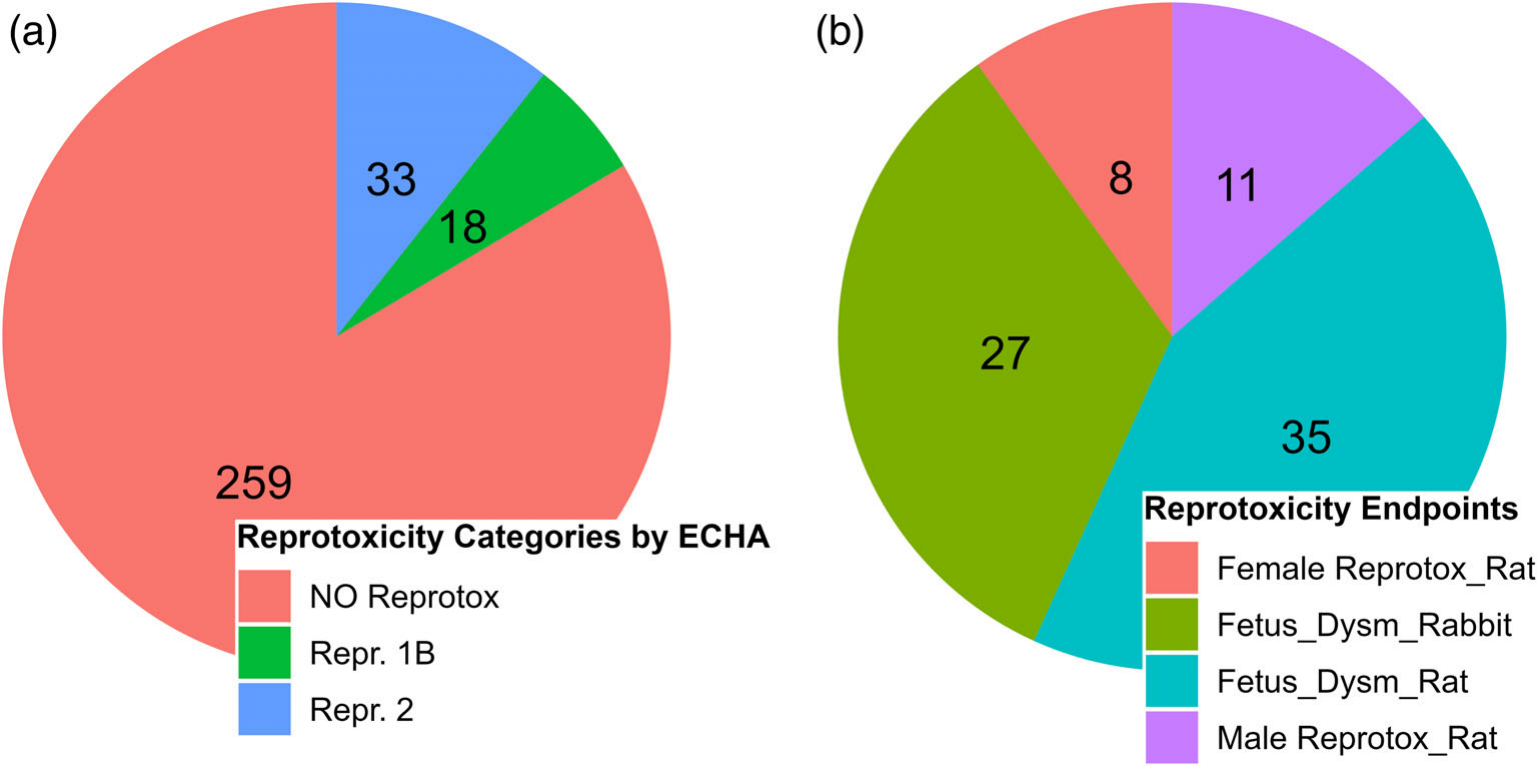
Pie charts of the distribution of (a) reprotoxicity categories by ECHA and (b) selected reprotoxicity endpoints within the pesticide DB. Pesticides classified as “NO Reprotox” do not have a Repr. 1A/B or 2 category classification but may have a classification for any other toxicity. The various reprotoxicity endpoints in chart B are based on the definition by Matthews et al. and were classified based on the studies relevant to the classification by ECHA (Matthews, Kruhlak, Daniel Benz, & Contrera, 2007)

To test the models with regard to their suitability for predicting reprotoxicity in pesticides, a database was created with 315 pesticides that were or are approved in the EU (see Tables S1 and S2 for pesticide DB). Five of these pesticides appeared in two versions each, which differed only in terms of stereoisomerism (cypermethrin, dimethenamid, cyhalothrin, napropamide, benalaxyl). Since most of the models to be tested do not differentiate between stereoisomers, only the 2D structures were considered in the evaluation (except for the OECD (Q)SAR Toolbox), which led to a database of 310 pesticides. In the database, the molecular structures were described by SMILES code and the InChIKeys. The reprotoxicity was assessed based on the ECHA classification according to CLP. Figure 1a shows the distribution of reprotoxicity due to ECHA classification. Notably, 256 pesticides were not classified as reprotoxicant. Notably, 17 were classified in Repr. Cat. 1B and 34 in Repr. Cat. 2. The relatively low number of potentially reprotoxic pesticides is explained by the fact that reprotoxicity is usually an exclusion criterion in the EU for the approval of a plant protection product. For the evaluation of the CAESAR model, the developmental toxicity was also selectively analyzed based on the hazard statements. Of the 51 pesticides classified as reprotoxic, only 5 are not developmentally toxic. Leadscope and CASE Ultra differentiate in their models between different endpoints of reprotoxicity: Female Reprotox_Rat, Male Reprotox_Rat, Fetus_Dysmorphogenesis_Rat, and Fetus_Dysmorphogenesis_Rabbit, which were defined by Matthews, Kruhlak, Daniel Benz, & Contrera (2007). The endpoints Female and Male_Reprotox_Rat include effects on the respective reproductive organs and fertility specific to the rat. Fetus_Dysmorphogenesis includes structural effects on fetal organs and tissues separated by species. Based on these definitions, the study results described in the available documents by ECHA/EFSA (RAC Opinion or Conclusion regarding the peer review of the pesticide risk assessment) were analyzed and corresponding columns added to the pesticide database to have comparable data. The number of pesticides per endpoint can be seen in Figure 1b. It is very important to mention, that the as reprotoxicants classified pesticides could show toxicity in one or many of these sections but also in none.

In addition, the categorization of the pesticides due to the different Resistance Action Committees (HRAC, FRAC and IRAC) and the BCPC's Compendium of Pesticide Common Names has been added, if available. The most common pesticide types in the database are fungicides, herbicides, and insecticides, a list of all types can be found in Table S3. Besides, all pesticides were classified based on their chemical structure (Chemical Group column). When categorizing according to these chemical groups, triazoles, sulfonylureas, carbamates, and organothiophosphates were the most common. Table 2 shows the 12 chemical groups with the most pesticides and the associated MOA according to the RAC poster, which applies to most of the categorized pesticides.

**TABLE 2:** The most common chemical groups within the pesticide DB with corresponding mode of actions by the RAC‐posters

Group	Pesticide type	Mode of action based on IRAC/FRAC/HRAC	#
Triazole	Fungicide	G1: Inhibition of sterol biosynthesis in membranes via C14‐ demethylase (19/21)	21
Sulfonylurea	Herbicide	2: Inhibition of acetolactate synthase (14/14)	14
Carbamate	Insecticide	1A: Acetylcholine esterase inhibitor (9/13)	13
Organothiophosphate	Insecticide	1B: Acetylcholine esterase inhibitor (10/10)	10
Pyrethroid	Insecticide	3A: Sodium channel modulator (9/9)	9
Aryloxphenoxypropionate (FOPs)	Herbicide	1: Inhibition of acetyl CoA carboxylase (7/7)	7
Phenoxycarboxylate	Herbicide	4: Auxin mimics (7/7)	7
Pyrazolecarboxamide	Fungicide	C2: Inhibition of succinate‐dehydrogenase (7/7)	7
Strobilurin	Fungicide	C3: Inhibition of cytochrome bc1 (ubiquinol oxidase) at Qo site (cyt b gene) (7/7)	7
Phenylurea	Herbicide	5: Inhibition of photosynthesis at PSll—serine 264 binders (5/7)	7
Chloroacetamide	Herbicide	15: Inhibition of very long‐chain fatty acid synthesis (6/6)	6
Dinitroaniline	Herbicide	3: Inhibition of microtubule assembly (5/6)	6

*Note*: The numbers in brackets indicate how many of the categorized pesticides can be assigned to the named mode of action. A list of all chemical groups can be found in Table S4

Abbreviations: FRAC, Fungicide Resistance Action Committee; HRAC, Herbicide Resistance Action Committee; IRAC, Insecticide Resistance Action Committee.

### 2.2 Used prediction models

In the following, a selection of commercial and freely available models for DART endpoints, which were used in the case studies (see Section 3), are introduced: the open source *in silico* tools *OECD (Q)SAR Toolbox* (v4.4.1, developed by Laboratory of Mathematical Chemistry (LMC), Bulgaria, in collaboration with the Organization for Economic Co‐operation and Development (OECD) and the European Chemicals Agency (ECHA), the *VEGA In Silico Platform* (v.1.1.5‐b48, developed by Istituto di Ricerche Farmacologiche Mario Negri [Laboratory of Environmental Chemistry and Toxicology] and Kode srl), the commercial software packages *Leadscope Model Applier* (v3.0.2‐4, developed by Instem), and *CASE Ultra* (v1.8.0.0, developed by MultiCASE Inc.). The predictions of all models and pesticides can be seen in Table S6.

#### 2.2.1 VEGA: Developmental Toxicity model (CAESAR, v.2.1.7)

The Developmental Toxicity CAESAR (Computer‐Assisted Evaluation of industrial chemical Substances According to Regulations) model is a QSAR classification model based on a random forest method implemented using WEKA open‐source libraries designed by Cassano et al., 2010. The underlying data set contains 292 compounds of different classes (extracted from Arena, Sussman, Mazumdar, Yu, & Macina, 2004) whose developmental toxicity was classified according to the FDA criteria and then was subdivided in two classes: nondevelopmental toxicant (N) (FDA Cat. A and B) and developmental toxicant (D) (FDA Cat. C, D, and X). Notably, 91 compounds were classified as non‐developmental and 201 as developmental toxicants. A set 13 descriptors was used for the description of the compounds calculated using Toxicity Estimation Software Tool (T.E.S.T.) (Cassano et al., 2010). The applicability of the CAESAR model is limited to organic substances with the usual elements. Predicting the toxicity of salts is only possible if they were converted into the neutralized form.

In addition to predicting toxicity using the CAESAR model, the VEGA platform itself provides an analysis of the AD. The AD is the structure space on which the training set of the model was developed and to which it is applicable to make predictions for new compounds (Hanser, Barber, Guesné, Marchaland, & Werner, 2019). To analyze the AD, the VEGA algorithm first determined the six most similar compounds within the training/test set of the model (Cassano & Benfenati, 2010). Chemical similarity was calculated by combining fingerprints with non‐binary structural keys based on constitutional molecular descriptors (Floris et al., 2014). Important is that this similarity calculation is completely independent from the CAESAR model itself. Then, the two most similar compounds were used to determine the AD index (ADI), which considers also other indices besides similarity. The ADI has values from 0 (worst case) to 1 (best case) and is the basis for the reliability classes good, moderate, and low. Since the training set is not very large, the provided information about the similar compounds and the ADI are very useful to evaluate the prediction.

The validation statistic states the sensitivity as 95% and the specificity as 59%. This is sensible since the models have been developed with the aim to minimize false negatives in order to make the CAESAR model usable for REACH (Cassano et al., 2010). This tendency has to be considered when analyzing the predictions.

#### 2.2.2 OECD (Q)SAR Toolbox

The expert‐based developmental and reproductive toxicity (DART) scheme (v.1.4, developed by Procter & Gamble and LMC) is based on a decision tree for identifying chemicals as developmental and/or reproductive toxicants presented in Wu et al., 2013. This decision tree was designed based on the combination of known modes of action (MOA) and associated structural features, as well as an empirical association of structural fragments within DART chemicals when MOA information was not available. According to Wu et al., 2013, the decision tree was not originally intended to be used as a standalone predictive tool, but as part of a screening system to identify potentially reproductively toxic chemicals and as part of weight‐of‐evidence‐based structure–activity relationship (SAR) decisions. This conflicts with use by the expert‐based DART scheme of the OECD (Q)SAR Toolbox.

The stereochemistry of the test substances is relevant to the prediction in this model, accepting the nine categories (2, 3, 4, 5, 6, 7, 14, 16, and 18) where stereoisomerism is ignored. Besides, the applicability of the model is limited to organic substances. The profiler's database comprises 716 chemicals (664 positive, 16 negative and 36 with insufficient data) that were investigated for their DART potential (OECD (Q)SAR Toolbox, 2020; Wu et al., 2013). It includes 25 different categories and 129 sub‐categories, based on defined receptor binding and chemical properties and, if known, their MOA. It should be noted that the tool is not intended as a stand‐alone system to support regulatory decision‐processes (OECD (Q)SAR Toolbox, 2020).

#### 2.2.3 VEGA: Developmental/Reproductive Toxicity library (PG, v.1.1.0)

The PG (Procter&Gamble) model is an empirically based decision tree designed by Wu et al., 2013 (see Section 2.2.2) and, therefore, is very similar to the DART model of the OECD (Q)SAR Toolbox. However, the PG model is available at the VEGA platform, which automatically calculates the most similar compounds of the training set, an applicability domain index (ADI) and based on this, indicates a reliability (Benfenati, 2020). This additional information is very helpful for assessing the prediction, for example, to check the classification in a certain category based on similar compounds. In this model, pesticides are predicted to be non‐toxic, if their core structural features fall outside of the chemical domains covered by the DART decision tree. It is important to realize that the PG model, by design, is incapable of predicting non‐reproductively toxic substances, as there are no such categories. The correct description would be that there is no known DART precedent, which does not automatically imply the absence of DART endpoint effects (Wu et al., 2013). As with the CAESAR model, the sensitivity here at 0.89 is significantly greater than the specificity at 0.44 (Benfenati, 2020).

#### 2.2.4 Leadscope Model Applier

The statistical models used in the Reproductive Toxicity Suite, Repro Female Rat (RFR) v2 and Repro Male Rat (RMR) v2, are intended to be used in screening, prioritization and can be used in a weight of evidence approach particularly for designing studies and interpretation of findings, which may be used in regulatory contexts (Leadscope, 2021). These models were developed under a Research Collaboration Agreement (RCA) with the United States Food and Drug Administration (FDA) (Leadscope, 2021). The training set of the RFR model consists of 894 structures and that of the RMR model consists of 714 (Leadscope, 2021), which were obtained from the Informatics and Computational Safety Analysis Staff (ICSAS) database described in Division of Applied Regulatory Science (DARS) publications of the FDA (Matthews et al., 2006a, 2006b). The training set of the Leadscope RFR model includes adverse effects on the female reproductive system and fertility, while it does not include effects on the fetus, gestation, or lactation. Reprotoxicity in the RMR model comprises adverse effects on the reproductive system and fertility in male rats (Matthews et al., 2006a). The RFR model comprises 14.08% positives in its training set, while the RMR model includes 30.07%. Because of the unbalanced nature of the training sets, each model combines the results of three sub‐models with balanced sets as average model (Leadscope development team, personal communication).

The structural features identified by the models are either positively or negatively correlated with activity. Such features are highlighted in the structure to facilitate a rapid review of features which are associated with activity and to assess the coverage of the structural elements by the models. This information is provided in the prediction report. The following eight property descriptors are used in the RFR and RMR models: A Log*p*, polar surface area, hydrogen bond acceptors, rotatable bonds, parent molecular weight, hydrogen bond donors, parent atom count, and Lipinski score (Leadscope development team, personal communication).

The Leadscope software uses the following parameters to manage the AD of the models: in addition to all property descriptors, at least one structural feature and at least one chemical in the training set with at least 30% global similarity to the test chemical is required to generate predictions (Leadscope, 2021). The similarity score is based on Leadscope's 27,000 sub‐structural features and hence will be lower than similarity scores that use smaller feature sets.

#### 2.2.5 MultiCASE Software

CASE Ultra is a commercial tool by MultiCASE, which provides classification models for different reproductive and developmental toxicity endpoints based on *in vivo* data for mouse, rat, or rabbit from FDA as part of Research Cooperation Agreement (RCA). Four of these endpoint models (see Table 3) were selected for evaluation of their predictive power for pesticides. The definition of these endpoint models was given by Matthews et al., 2007 and the pre‐processing of data before modeling was published in Matthews et al., 2006a. The models were based on different data sets, but all were statistical based SA models, which were built by collecting positive or deactivating alerts from the training data set that are related to the toxicity being modeled (Chakravarti et al., 2012). In addition, a local QSAR was built for each alert with physicochemical descriptors. The outcome of the prediction was given as the probability of being reprotoxic on a scale of 0 to 1 and by use of the classification threshold (CT) (specific for each model) the prediction was done. A probability between 0 and CT−0.1 leads to a negative or out of domain classification. When the probability is between CT−0.1 and CT+0.1 the substance is classified as inconclusive and above CT+0.1 as positive. The AD of the model is defined by a fragment based chemical space defined by the training set chemicals (Cioffi, 2019a, 2019b, 2019c, 2019d). The AD is assessed by checking for 3‐atom fragments that are not present in the trainings set. Due to the limits of applicability inorganic compounds, mixtures and large biomolecules are in principle not covered by the AD. The prediction report provided by CASE Ultra contains detailed information about the alerts and structural analogs etc., which are of great importance when assessing the prediction.

**TABLE 3:** The properties of the evaluated CASE Ultra models

Model	Description	Species	# Active/inactive	# Descriptors	Classification threshold
FDYSM	Fetal Dysmorphogenesis	Rabbit	128/129	19	0.5
		Rat	436/457	111	0.45
FFRET	Female fertility	Rat	113/113	47	0.55
MFRET	Male fertility	Rat	180/180	47	0.5

### 2.3 Evaluation of predictions

Analysis was conducted in KNIME (version 4.3.2) (Berthold et al., 2008) and R (version 4.0.2) (R Core Team, 2019) and figures were produced using the R package ggplot2 (Wickham, 2016). All shown chemical structures were copied from PubChem or from the respective model reports. For the assessment, the predicted toxicity of the PG and the QSAR Toolbox model was compared with the classification by the ECHA. In the case of the CAESAR model, predictions were compared to developmental determined by ECHA. For the Leadscope and CASE Ultra models, the results of the animal experiments in rats and rabbits on which the ECHA classification is based were used. If the pesticide was predicted as nontoxic the evaluation could be True Negative (TN) or False Negative (FN) and if the prediction was toxic the possible evaluations were True Positive (TP) or False Positive (FP). In the case that no reliable prediction could be made the evaluation is UNKNOWN (see Table 4).

**TABLE 4:** List of possible predictions of all models and the resulting evaluations

Model	Prediction	Evaluation
VEGA_CAESAR	NON‐toxicant (experimental value, good/moderate reliability)	TN, FN
	Toxicant (experimental value, good/moderate reliability)	TP, FP
	NON‐toxicant/toxicant (low reliability)	UNKNOWN
VEGA_PG	NON‐toxicant	TN, FN
	Toxicant	TP, FP
OECD (Q)SAR Toolbox (OQTB)	Not known precedent reproductive and developmental toxic potential	TN, FN
	Known precedent reproductive and developmental toxic potential	TP, FP
	Not covered by current version of the decision tree	UNKNOWN
Leadscope (LS)	Negative/Negative_EV	TN, FN
	Positive/Positive_EV	TP, FP
	Missing descriptors/not in domain	UNKNOWN
CASE Ultra (CU)	Negative/known negative	TN, FN
	Positive/known positive	TP, FP
	Inconclusive/out of domain	UNKNOWN

Abbreviations: FN, false negative; FP, false positive; TN, true negative; TP, true positive.

Besides the typical values of an error matrix (TN, FN, TP, FP), also the sensitivity (SEN), specificity (SPC), accuracy (ACC), and balanced accuracy (BA) were determined (for definitions see Table 5). For models that predict toxicity then sensitivity is a more critical value than the specificity, because of safety reasons FP are much more tolerable than FN. In this study, the pesticide DB and also most of the training sets of the models were unbalanced data sets; therefore, the BA is calculated besides the more common ACC.

**TABLE 5:** The formulas for calculating the typical parameters to evaluate prediction models

Value	Name	Definition
SEN	Sensitivity	TPTP+FN
SPC	Specificity	TNTN+FP
ACC	Accuracy	TP+TNTP+TN+FP+FN
BA	Balanced accuracy	SEN+SPC2

Abbreviations: FN, false negative; FP, false positive; TN, true negative; TP, true positive.

## 3 RESULTS AND DISCUSSION

### 3.1 CAESAR model of VEGA

The statistical developmental toxicity QSAR model CAESAR is based on a data set of 292 compounds whereby the majority of the compounds was classified as “Toxicant” (69%). The model is available via the VEGA platform, which provides an assessment of the reliability in addition to the prediction. This reliability relates to whether the connection is inside or outside the model's AD.

#### 3.1.1 Evaluation

Table 6 illustrates the reliability distribution within the tested pesticide database. The vast majority of pesticides were outside the AD of the model (77%). This shows that the model cannot provide a meaningful prediction for most pesticides.

**TABLE 6:** The distribution of reliability of the developmental toxicity prediction of 310 pesticides using the CAESAR model provided by VEGA

Reliability	Applicability domain	#	# [%]
Experimental value	The predicted compound *could be out* of the Applicability Domain of the model	1	0.32
Good reliability	The predicted compound *is into* the Applicability Domain of the model	28	9.03
Moderate reliability	The predicted compound *could be out* of the Applicability Domain of the model	40	12.90
Low reliability	The predicted compound *is outside* the Applicability Domain of the model	241	77.74

The predictions were evaluated by comparing them with the GHS classification of ECHA referring to developmental toxicity (see M&M). Pesticides whose prediction was classified as unreliable (Out of AD) were classified as UNKNOWN for the evaluation. This resulted in a large number of false positives, especially for the pesticides, the prediction of which was classified as good (see Table 7). This observation agrees with the results of the published validation of the model (Cassano & Benfenati, 2010), which also show a high proportion of false positives and thus a low specificity. This is due to the high overhang of toxic compounds in the training data set of the CAESAR model and is reinforced by the opposite distribution in the pesticide database. The proportion of false negatives, on the other hand, is very low, which leads to a sensitivity of 0.89.

**TABLE 7:** The results of evaluation of the CAESAR model via typical parameters

	# FN	# FP	# TN	# TP	# UNKNOWN	SEN	SPC	BA	ACC
ALL	2	42	8	17	241	0.89	0.16	0.53	0.36
Experimental value	0	0	1	0	‐	‐	1.00	‐	1.00
Good reliability	1	23	1	3	‐	0.75	0.04	0.40	0.14
Moderate reliability	1	19	6	14	‐	0.93	0.24	0.59	0.50

*Note*: In addition to the evaluation for all pesticides, the following lines contain the evaluation related to the prediction reliability.

Abbreviations: ACC, accuracy; BA, balanced accuracy; FN, false negative; FP, false positive; SEN, sensitivity; SPC, specificity; TN, true negative; TP, true positive.

#### 3.1.2 Example

The following example shows in detail why false predictions are made despite good reliability (ADI > 0.8): Napropamide is an herbicide that belongs to the chemical group of acetamides. According to the GHS classification, napropamide is not toxic to development, but was classified as developmental toxicant by the CAESAR model, therefore as a false positive prediction. The reliability was given as good (ADI = 0.918), which means that napropamide was within the AD of the model. This classification is based on the two most similar compounds in the training data set of the model and their classification, which can be viewed in the report. The two most similar substances were Phenyltoloxamine and Naproxen with a similarity score of 0.855 and 0.83 (see Table 8). According to the model, connections with similarity scores above 0.75 are to be regarded as sufficiently similar. This seems questionable when comparing the chemical structure of napropamide with phenyltoloxamine and naproxen. The main structures of napropamide methoxynaphthalene and acetamide were not mirrored. In addition, phenyltoloxamine was only part of the test set and therefore did not serve as the basis for the model. Overall, similarity scores should be viewed critically, since the values depend heavily on the choice of descriptors, as can be seen in Table 8. Next, the toxic classification of the similar compounds is considered. Both were classified as toxic to development due to their FDA classification, while the ECHA only classifies naproxen as developmentally toxic.

**TABLE 8:** Example for a false positive CAESAR prediction despite good reliability

	Name	Structure	Developmental toxicant?	CAESAR prediction	Similarity by VEGA	Average Tanimoto similarity coefficient			
						Pub chem	RDKit	Morgan	Feat Morgan
Tested pesticide	Napropamide	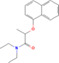	NO (ECHA)	Toxicant		1	1	1	1
Similar compound 1	Phenyltoloxamine	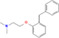	YES (CAESAR)	Toxicant	0.855	.828	.345	.208	.205
Similar compound 2	Naproxen	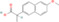	YES (CAESAR)	Toxicant	0.83	.671	.331	.212	.282

*Note*: Napropamide was the tested pesticide and phenyltoloxamine and naproxen were the most similar compounds of the data set of the CAESAR model. To show the variability of similarity depending on the selected descriptors, the similarity score provided by the VEGA platform was compared with the average Tanimoto similarity coefficient based on different fingerprints.

#### 3.1.3 Summary

In summary, many wrong predictions despite good reliability were made because of an insufficiently similarity of the “most similar compounds” as well as different data sources for the assessment of toxicity. Therefore, the similarity of the compounds and the data sources should always be checked when assessing the prediction.

### 3.2 PG model of VEGA

The PG model for the prediction of DART is available via the VEGA platform. It is a rule‐based model where the classification takes place via a decision tree. The compounds are categorized into 25 different chemical categories including several subgroups. It is important that the established rules are only suitable for the detection of DART, but that there are no rules that describe non‐DART structures. Therefore, the consideration of reliability only makes sense for categorized and thus classified as toxic pesticides, since all others should not be within the AD of the model by definition (see Table S8).

#### 3.2.1 Evaluation

When analyzing the results, a large number of pesticides (39, 12.5% of all pesticides, see Table S8) were labeled as experimental value, which means that they can also be found in the training data set of the model. Of these, 59% were false positive, which indicates a different interpretation of DART in the data set of the PG model and by the ECHA (see Table 9).

**TABLE 9:** The results of evaluation of the PG model via typical parameters

	# FN	# FP	# TN	# TP	SEN	SPC	BA	ACC
ALL	34	77	182	17	0.33	0.70	0.52	0.64
Experimental value	0	23	3	13	1.00	0.12	0.56	0.41
Categorized	0	77	0	17	1.00	0	0.50	0.18
Uncategorized	34	0	182	0	0	1.00	0.50	0.84

*Note*: In addition to the evaluation for all pesticides, the following lines differentiate between experimental value and categorized or uncategorized pesticides.

Abbreviations: ACC, accuracy; BA, balanced accuracy; FN, false negative; FP, false positive; SEN, sensitivity; SPC, specificity; TN, true negative; TP, true positive.

**FIGURE 2:**
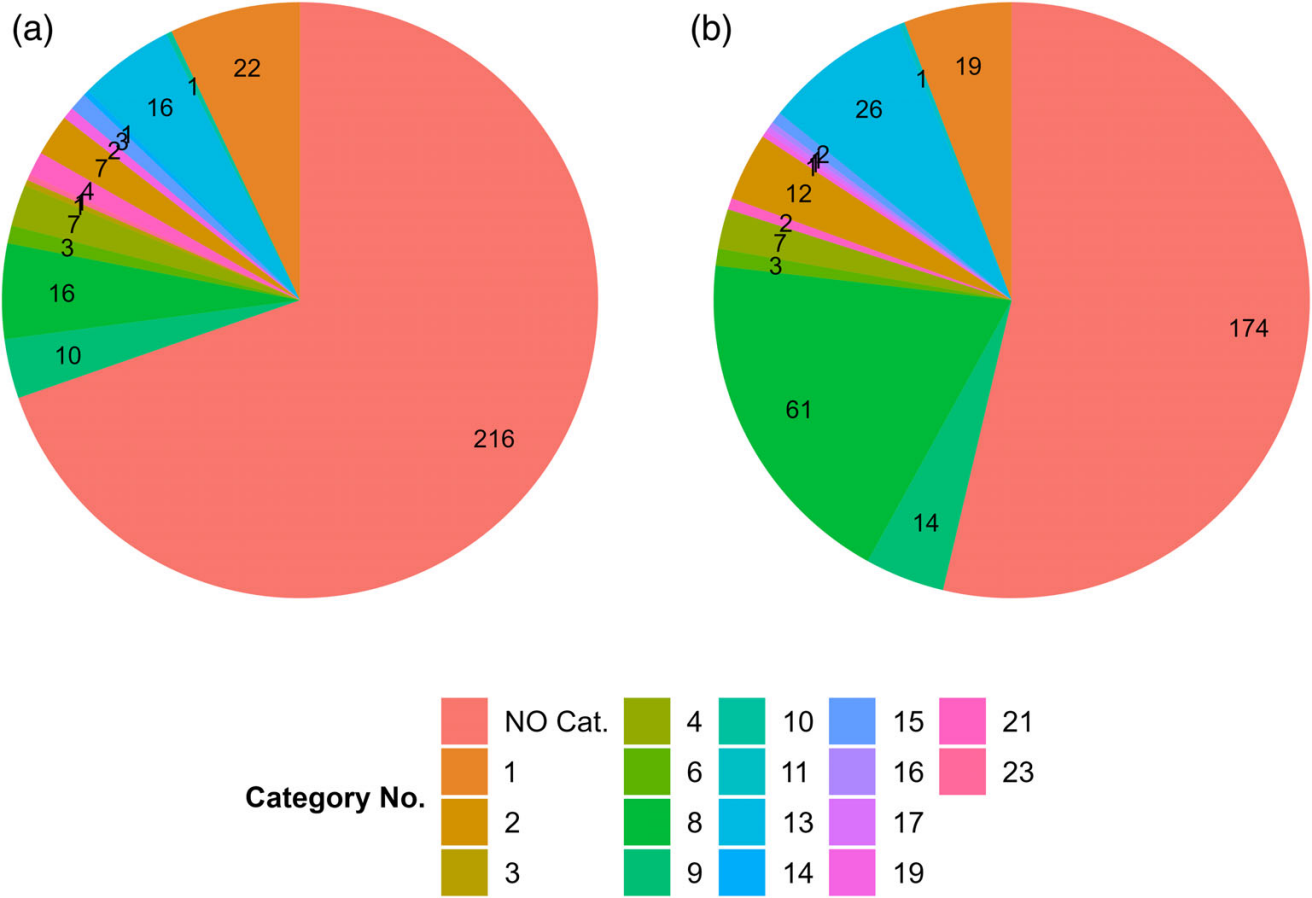
The pie charts show the distribution of pesticides in the chemical categories defined by Cassano et al. (2010) predicted by the PG model (a) or DART scheme of the OECD (Q)SAR Toolbox (b). The structural description of the categories can be found in Table S7

In predicting the DART of the pesticide database, 216 pesticides were not categorized and thus classified as non‐toxic. All other categorized were divided into 14 categories, with categories 1 (inorganics and derivatives metals, metallic derivatives, organophosphorus and organosiloxane compounds), 8 (aromatic compounds with alkyl, multi‐halogen and nitro groups), and 13 (imidazole, nitro imidazoles derivatives, nitro‐furfurylideneamino and triazole derivatives) being the most common (see Figure 2a and Table S9).

**FIGURE 3:**
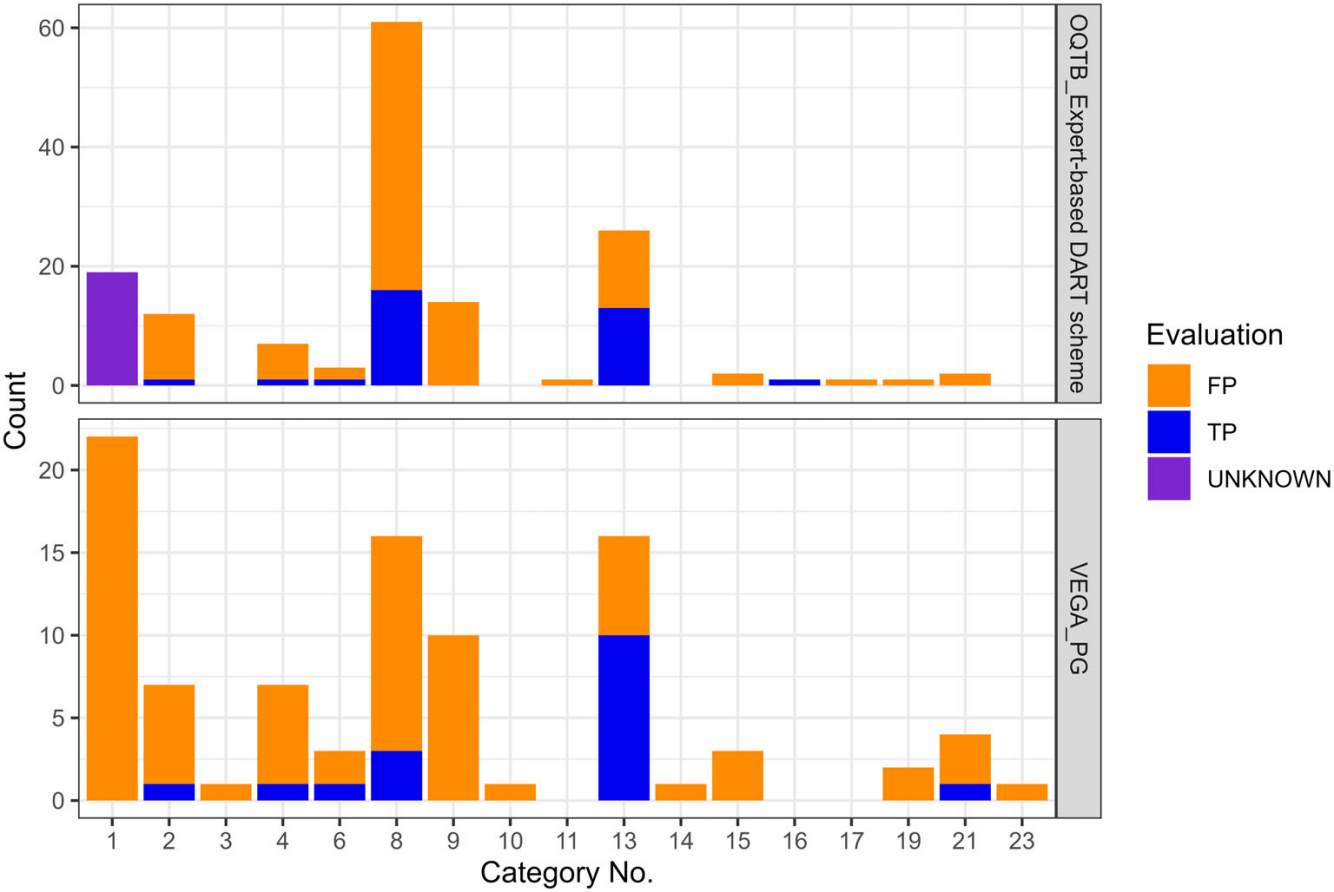
The bar plots show the evaluation of the predictions divided by the predicted categories for the PG and DART model by OECD (Q)SAR Toolbox. The aim of the depiction is to analyze whether the prediction for some categories is more reliable than for others. FP, false positive; TP, true positive

Overall, the model classified two‐thirds of the pesticides that are toxic to reproduction as non‐toxic (see Table 9). These belonged to different chemical groups and were either not assigned to the “right” category or there was no suitable category. The proportion of false positives was 25%. Figure 3 shows the distribution of FP and TP per category. In all categories, the number of FPs was higher than the number of TPs except for category 13, which includes triazole and imidazole.

#### 3.2.2 Example

In the following, the evaluation of the prediction is shown on the basis of the PDF report provided by VEGA using the example of 2,4‐dichlorophenoxyacetic acid (2,4‐D) (see Table 10). 2,4‐D is a phenoxy herbicide belonging to the auxins group. It was classified as toxicant due to experimental value, which was different to the ECHA classification. 2,4‐D was categorized into category 8c (aromatic compounds with alkyl, multi‐halogen and nitro groups, examples: para‐dichlorbenzene, 1,2,4‐trichlorobenzene) based on the dichlorobenzene sub‐structure (matching rule/virtual compound, see Table 10). The phenoxy acetic acid part was not taken into account, which leads to a misleading categorization. All six most similar compounds provided by the VEGA software were phenoxy herbicides and categorized to category 9c (alpha aryloxy substituted acetic acid, examples: 2,4,5‐trichlorophenoxyacetic acid, 2,4‐D Isopropyl ester), which would to be a much more suitable category also for 2,4‐D. Since the prediction was false positive, although 2,4‐D is part of the training data set of the PG model, the data source was of great interest. The DART toxicity of 2,4‐D was described by the Reproductive respectively Developmental Toxicity Effect Codes R(T) (Changes in reproductive function/fertility only occurred at doses where there was significant toxicity on other organ systems) and D(MT) (Developmental effects only occur in the presence of maternal toxicity) and the Reregistration Eligibility Decision document by U.S. EPA was given as reference U.S. EPA, 2005a, 2005b. In this case, the problem lies in the fact that the classification is based on different study data, or the study data were interpreted differently.

**TABLE 10:** Structure of the tested pesticide 2,4‐D, the matching rule/virtual compound and the two most similar compounds, as well as their predicted categories by the PG model

	Name	Structure	Predicted category
Tested pesticide	2,4‐D	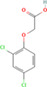	8c
Matching rule/virtual compound	‐	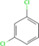	‐
Similar compound 2	2,4,5‐trichlorophenoxyacetic acid	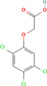	9c
Similar compound 3	2,4‐D isopropyl ester	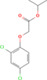	9c

#### 3.2.3 Summary

The PDF report of the PG model describes exactly based on which structure fragment the pesticide was classified in the respective category. The more structures of the original molecule are covered, the better. In contrast to most models, the PG model also offers a detailed description of the sources on the basis of which the compounds in the data set were classified. All of this information should be considered when assessing the prediction.

### 3.3 DART scheme of OECD (Q)SAR Toolbox

The aim of the Developmental and Reproductive Toxicity (DART) scheme implemented in the OECD (Q)SAR Toolbox is to indicate that the test compound is associated with chemical structures known to have DART, or that it contains structural features that are outside the AD of the DART decision tree (OECD (Q)SAR Toolbox, 2020).The Toolbox's DART scheme is a rule‐based profiler in which the classification is carried out using a decision tree (OECD (Q)SAR Toolbox, 2020), similar to the PG model. This decision tree includes 25 different chemical categories including 129 subcategories (OECD (Q)SAR Toolbox, 2020). It should be noted that the established rules are only suitable for the detection of DART, but there are no rules that describe non‐DART structures. In contrast to the PG model, with the Toolbox's DART scheme there is no structure‐based comparison of the predicted substance with the training data set and therefore no evaluation of the AD.

The pesticides were classified into one of the following three categories: “Not known precedent reproductive and developmental toxic potential,” “Known precedent reproductive and developmental toxic potential,” or “Not covered by current version of the decision tree.” The latter category means that the test compound is out of AD of the DART profiler. Thus, such compounds are not classified by the DART scheme (OECD (Q)SAR Toolbox, 2020). In addition, the following subcategories were identified for the pesticides tested in this category: “Inorganic chemical,” “Metal atoms were identified, Metals (1a),” or “Organophosphorus compounds (1b).” For the evaluation of the pesticide predictions, the category that was identified outside of the AD is interpreted as UNKNOWN. Further, if a substance does not match one of the structural features associated with the potential to act as a DART compound, it is classified as “Not known precedent reproductive and developmental toxic potential.”

In contrast to the other models, the DART model of the OECD (Q)SAR Toolbox distinguishes between stereoisomers, which is why these were also used for the prediction when relevant. There were no differences in the prediction between the stereoisomers of the tested pesticides or in comparison with the 2D structures.

#### 3.3.1 Evaluation

Most pesticides (56%) were not associated with chemical structures known to have DART and were therefore identified as negative, 37% were predicted as positive, and 6% were outside of the profiler's AD. In comparison, the similar PG model implemented in the VEGA platform predicted 70% of the pesticides as negative, 30% as positive, and 0% were outside the AD of the model.

In the DART prediction of the pesticide database, a total of 136 categorized pesticides (117 DART positives and 19 compounds that were outside the AD) were divided into 13 categories (Figure 2b) and 18 subcategories. Of these 150 pesticides, 14 were categorized into two categories. The higher proportion of 174 pesticides was not assigned to any category (56%) and was therefore predicted as negative. Table S10 shows the distribution of pesticides in the respective categories and subcategories.

In the following, either all prediction results of the Toolbox's DART scheme or only uncategorized and categorized results are examined (Table 11) and compared with VEGA's PG model.

**TABLE 11:** The results of evaluation of the DART scheme of the OECD (Q)SAR Toolbox via typical parameters

	# FN	# FP	# TN	# TP	# UNKNOWN	SEN	SPC	BA	ACC
ALL	22	88	152	29	19	57	63	60	62
Categorized	0	88	0	29	0	100	0	50	25
Uncategorized	22	0	152	0	0	0	100	50	87

*Note*: In addition to the evaluation for all pesticides, the following lines differentiate between categorized and uncategorized pesticides.

Abbreviations: ACC, accuracy; BA, balanced accuracy; FN, false negative; FP, false positive; SEN, sensitivity; SPC, specificity; TN, true negative; TP, true positive.

When investigating all of the pesticide predictions, the Toolbox's DART profiler predicted 43% of the pesticides that are DART positive as non‐toxic compared to the ECHA GHS classification. This is a better prediction result compared to the similar PG model, which classified 66% as non‐DART. This group of false negative tested pesticides similarly includes different pesticide types and chemical groups in both models. The false negatives were either not classified to the “right” category or there was no appropriate category. A sensitivity of 57% and a specificity of 63% were identified in the Toolbox's DART scheme, while a sensitivity of only 33% and a specificity of 70% were revealed in the PG model.

Of the 174 uncategorized pesticides identified as negative in the Toolbox were 87.4% classified as true negative and 12.6% as false negative. The PG model showed similar results, in which 216 uncategorized pesticides were identified 84.3% as true negative and 15.7% as false negative.

More pesticides were classified in the Toolbox than in the PG model. Of the 117 categorized DART positives in the Toolbox were 24.8% classified as true positive and 75.2% as false positive, while of the 94 classified DART positives in the PG model were 18.1% identified as true positive and 81.9% as false positive. The distribution of true positive and false positive predictions per chemical category in the Toolbox is presented in Figure 3. In all categories with a higher number of pesticides (i.e., ≥ 7 pesticides/category), the number of false positives was higher than that of true positives, with the exception of Category 13, which included both with the same frequency. Category 13 includes triazole and imidazole. These results are similar to those of the PG model with the exception that more true positives were recognized in Category 13. Further, as mentioned above, more pesticides were categorized using the Toolbox's DART scheme than the PG model. In particular, the number of pesticides in chemical Category 8 (above all “Toluene and small alkyl toluene derivatives (8a)” and “Polyhalogenated benzene derivatives (8c)”) was much higher with the Toolbox's DART profiler than with the PG model (Figure 3).

It should be kept in mind that both models contain unbalanced training sets, with 92.7% positives, only 2.2% negatives, and 5% substances with insufficient data in their databases (OECD (Q)SAR Toolbox, 2020; Wu et al., 2013). Only DART positive structural alerts are used to categorize the test substances. This strong imbalance in the direction of DART positives in the training set may cause the high number of false positive results.

In general, the Toolbox's DART profiler has a slightly better statistical profile in terms of DART prediction compared to the VEGA's PG model. However, both DART models tend to predict a higher number of false positives and therefore show low specificity. Hence, both systems are “overcautious” and may hinder the regulatory decision‐process of pesticides.

One of the model differences is that the Toolbox's DART profiler classifies all pesticides of Category 1 (“Inorganic chemical,” “Metal atoms were identified, Metals (1a),” and “Organophosphorus compounds (1b)”) as “Not covered by current version of the decision tree” (UNKNOWN; Figure 3), while the pesticides in Category 1 (“Inorganics and derivatives: metals, metallic derivatives, organophosphorus, and organosiloxane compounds”) of the PG model are assigned as toxicants. However, when comparing the Category 1 pesticides of both models with the ECHA GHS classification, none of them were classified as DART positive. It can therefore be concluded that an incorrect classification was implemented in the PG model for Category 1 substances.

A more detailed comparison of the predictions of both models shows that of the 310 pesticides tested, 157 were not categorized by both models, 66 were assigned similarly, and only 2 pesticides (1,4‐dimethylnaphthalene and 2,4‐D) were classified in different categories. Further, 54 were only categorized by the DART profiler of the Toolbox and 17 only by the PG model (see Figure S1A). Of the 54 pesticides categorized only by the Toolbox's DART profiler, most were classified as Category 8 (“Toluene and small alkyl toluene derivatives (8a)”: 29 and “Polyhalogenated benzene derivatives (8c)”: 10) and 13 (“Triazole derivatives (13c)”: 10) (Figure S2A). In contrast, of the 17 pesticides that were only categorized by the PG model, most of them were assigned to Category 1 (“Inorganics and derivatives: metals, metallic derivatives, organophosphorus and organosiloxane compounds”: 4) and 8 (“Aromatic compounds with alkyl, multi‐halogen, and nitro groups”: 3) (see Figure S2B). In addition, the Toolbox's DART profiler assigned 14 pesticides to 2 categories, 5 (e.g., fluquinconazole) of which were only categorized by the DART profiler, for 8 (e.g., penconazole) one categorization was similar to the PG model and the other was not and for 1 (dicloran) both categories of the DART profiler were similar to the PG model (Figure S1B).

#### 3.3.2 Example

In a group‐based case study, the classification of DART positive pesticides in the Subcategory “Toluene and small alkyl toluene derivatives (8a)” by the Toolbox's DART profiler is investigating in the following.

**FIGURE 4:**
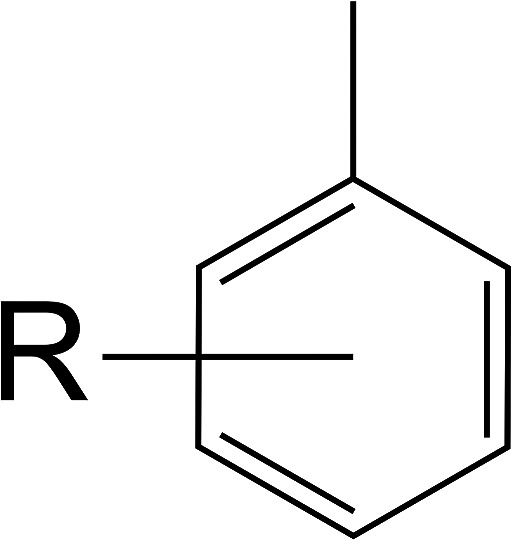
The structural scope of “Toluene and small alkyl toluene derivatives (8a).” R = H, Me, nBu, iPropyl, tBu

The structural framework of this subcategory implemented in the Toolbox is presented in Wu et al., 2013 and further developed by Procter & Gamble and LMC, Bulgaria (OECD (Q)SAR Toolbox, 2020). Only toluene and a single attached alkyl chain substituent (< 5 carbon atoms) are structural features of this category according to Wu et al., 2013 (Figure 4). The possible alkyl chain substituents can be at ortho‐, para‐ or meta‐positions. Members of the training data set (e.g., toluene, p‐xylene or butyltoluene) meet these conditions (Wu et al., 2013).

It is noticeable that all 29 pesticides assigned to Subcategory 8a by the Toolbox contain, in addition to toluene, larger substitutes (> 5 carbon atoms; including N, O, Cl, F, S, or Br atoms) that are not described in the original category definition of Wu et al., 2013 (selected pesticides shown in Table 12). Therefore, the categorization in Subcategory 8a is considered wrong, since the pesticides do not belong to the chemical class of toluene and small alkyl toluene derivatives. The similar PG model, on the other hand, which is closer to the description of Wu et al. (2013), did not classify any of the pesticides in Subcategory 8a.

**TABLE 12:** A selection of pesticides that were incorrectly classified in subcategory 8a

Name	1,4‐dimethyl‐naphthalene	Bifenthrin	Cyazofamid	Iprovalicarb	Metrafenone
CAS no.	571‐58‐4	82657‐04‐3	120116‐88‐3	140923‐17‐7	220899‐03‐6
Structure	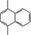	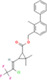	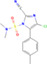	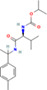	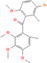

In conclusion, the classification of the 29 pesticides in Subcategory 8a is overall wrong or is not based on the requirements described in Wu et al., 2013. Hence, the Toolbox's DART profiler is not reliable to predict the DART potential of pesticides that contain toluene and alkyl toluene derivatives.

#### 3.3.3 Summary

The case study with toluene and alkyl toluene derivatives illustrates well the general problem of the QSAR prediction for pesticides using the Toolbox's DART scheme. Many false negative and false positive predictions were generated with the Toolbox, probably mainly due to incorrect classification of pesticides into different chemical categories. Therefore, when evaluating the predictions, care should be taken to ensure that the categorization of the chemical classes is correctly chosen by the Toolbox.

### 3.4 Leadscope

In the present publication, prediction results from Repro Female Rat (RFR) and Repro Male Rat (RMR) statistical QSAR models of the Reproductive Toxicity Suite were analyzed. The training set of the Leadscope RFR model includes only adverse effects on the female reproductive system and fertility, while it does not include effects on the fetus, gestation, or lactation. Reprotoxicity in the RMR model only comprises adverse effects on the reproductive system and fertility in male rats (Matthews et al., 2006a). Therefore, the predictions were compared with the results of the experiments relevant for the classification according to ECHA, based on the endpoints mentioned.

Both QSAR models assess potential reprotoxicity of test substances based on a statistical weighting of structural features present in the test structures as well as whole molecule descriptors. If experimental data are available within the Reproductive Toxicity Suite, these data will be used instead of the QSAR prediction. Probability scores below the cut off value of .5 are negative and values equal to or greater than .5 are considered positive (see Figure S3).

#### 3.4.1 Evaluation

When analyzing the prediction results of both models (Table 13), about half of the pesticides were classified as “UNKNOWN,” which comprises “Missing Descriptors” and “Not in Domain” calls (RFR: 47%, RMR: 59%) (see Table S11). A “Not in Domain” call indicates that the predicted pesticides were outside the model's AD. In the case of “missing descriptors,” this is due to inorganic structures for which the whole molecule descriptors cannot be calculated. Due to this classification, 4 reprotoxicants and 143 non‐reprotoxicants could not be predicted by the RFR model, and 4 reprotoxicants and 179 non‐reprotoxicants were not recognized by the RMR model when the predictions were compared with the ECHA GHS classification. Thus, 50 or 36% of the reprotoxicants could not be detected for each model, as they were outside the AD.

**TABLE 13:** The results of evaluation of the two Leadscope models Repro Female Rat (RFR) and Repro Male Rat (RMR) via typical parameters

Model	# FN	# FP	# TN	# TP	# UNKNOWN	SEN	SPC	BA	ACC
RFR	3	3	156	1	147	0.25	0.98	0.62	0.96
RMR	4	26	94	3	183	0.43	0.78	0.61	0.76

Abbreviations: ACC, accuracy; BA, balanced accuracy; FN, false negative; FP, false positive; SEN, sensitivity; SPC, specificity; TN, true negative; TP, true positive.

The Leadscope RFR and RMR models contain experimental data of the Informatics and Computational Safety Analysis Staff (ICSAS) database (Matthews et al., 2006a) with data records from FDA segment I (reprotoxicity in male and female rats) studies. The data were obtained from publicly available sources (e.g., Shepard's Catalog of Teratogenic Agents, TERIS, REPROTOX, and RTECS), as well as studies reported in drug labeling (Matthews et al., 2006a).

When comparing the prediction of pesticides, which are not only available in the pesticide database, but also in the training set, it is noticeable that these pesticides with experimental data are also sometimes “incorrectly” predicted (see Table S12). This is due to differences in the data from the Reproductive Toxicity Suite and the ECHA data set. The reason for these differences can be, for example, different *in vivo* studies on which the assessment is based or different evaluation of reprotoxic effects as adverse or not adverse.

Both models show a low sensitivity (RFR: 0.25, RFM: 0.43) in predicting the specific reprotoxicity endpoints of pesticides, while the specificity is high (RFR: 0.96, RFM: 0.76) (Table 13). If the statistical profile of the pesticide predictions is compared with those of the organic chemicals in the Leadscope manual, the sensitivity for the organic chemicals is much higher in both models (RFR: 61%, RFM: 85%), while the specificity is comparable (RFR: 95%, RFM: 73%) (Leadscope, 2021). The low sensitivity of both Leadscope models confirms that they should not be used in isolation for the regulatory evaluation of pesticides and additional lines of evidence such as through an expert review of model features and potentially reactive features, a consensus approach using predictions from other models in the Reproductive Toxicity Suite and/or experimental findings are needed.

As mentioned above, structural features and property descriptors are used to determine a probability score that drives the prediction. The distribution of probability scores per prediction can be seen in Figure S3. It is crucial for the assessment of the prediction, that the positive or negative statement is based on a threshold value (0.5) and a connection with the stability of the prediction and the absolute value of the probability cannot be considered without other information.

#### 3.4.2 Examples

In a group‐based case study, the prediction results of the structurally diverse conazole fungicides (imidazoles and triazoles) from the two selected RFR and RMR models were analyzed (see Table S13). Conazoles, a class of azole‐based fungicides, are widely used as pesticides, but also as human pharmaceuticals to treat mycoses (Kjærstad, Taxvig, Nellemann, Vinggaard, & Andersen, 2010; Zarn, Brüschweiler, & Schlatter, 2003) during pregnancy (King, Rogers, Cleary, & Chapman, 1998; Mogensen et al., 2017).

Of the 24 conazole fungicides included in the EFSA conclusions, two substances (epoxiconazole and triadimenol) are reprotoxic in female rats and one (triadimenol) in male rats according to their ECHA GHS classification. However, only one of the tested conazoles (i.e., epoxiconazole) was correctly predicted as reprotoxic by the RFR model, while the RMR model identified the substance false positive. The other reprotoxic pesticide, triadimenol, was either classified as false negative in the female model or outside the AD in the male model.

The negative prediction of triadimenol by the RFR model, due to the detected structural feature contribution of benzene, 1‐alkoxy, 4‐chloro (Table 14) and the property descriptors, which resulted in a probability score of .113, was evaluated as a false negative. The poor coverage of the structure by the feature identified indicates that an expert review of the prediction is necessary. An expert review may consider the training set structures, which map to the feature, potentially reactive features, and analogous structures. Analogous structures with a similarity score greater than 30% are indicated. Of these, it is important to examine the analogs and identify if any (based on structural or biological similarity) would be relevant for assessing the validity of the model prediction. The analog field contains two conazoles (croconazole and fluconazole, see Table 14). Based on representation from the same class, these analogs would be considered useful for further analysis. Croconazole is indicated as positive for adverse effects to female reproductive organs and fertility, while fluconazole is negative for these effects. Accessing the underlying data for fluconazole indicates result findings of specific developmental abnormalities to the central nervous system, craniofacial, and musculoskeletal system (Lopez‐Rangel & Van Allen, 2005). Such information may alert the reviewer to the lower reliability of the negative prediction and may support overturning the prediction based on review findings. The RMR model identified a kind of chlorophenol feature as a mitigating structural feature (Table 14), but no analog with at least 30% global similarity to triadimenol could be detected by the model. Therefore, the RMR model considered the pesticide to be outside the AD.

**TABLE 14:** Detected structural features and selected training set analogs of triadimenol, which is reprotoxic in female and male rats

Predicted pesticide	Model	Evaluation	Detected structural features	Selected relevant analog structures	
*Triadimanol* CAS no. 55219‐65‐3 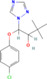	RFR v2	FN	Benzene, 1‐alkoxy‐, 4‐chloro‐ 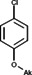	*Croconazole* *Positve* for RFR 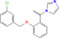	*Fluconazole* *Negative* for RFR 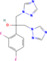
	RMR v2	UNKNOWN	Chlorophenol‐ 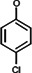	No analog structures reported.	

For epoxiconazole, the true positive classifications by the RFR model and false positive classification by the RMR model were based on evidence of structural feature contributions (RFR: benzene, 1‐halo, 4‐oxymethyl‐feature, RMR: Fluorobenzene structure represents one of four identified features, see Table 15) and property descriptors associated with the predicted specific effect. Given the totality of positive/negative contributing traits in the pesticide structure, the positive probability for reprotoxicity in both models was above the cut‐off: the RFR model identified a positive probability of .614 for the true‐positive prediction and the RMR model for the false positive result was .514, which is slightly above the cut‐off positive prediction by both Leadscope models. The structural similarity of the analogs with epoxiconazole was between 32 and 39% in both models. Looking at the identified analogs, it is striking that of the 7 (RFR) or 6 (RMR) conazole analogs, only one is positive for the respective specific toxicity (see Table 15). This could mislead to the unreflecting assumption that both predictions are wrong, although this is only true for the RMR prediction. Therefore, this information must be carefully considered in the context of an expert opinion.

**TABLE 15:** Detected structural features and selected training set analogs of epoxiconazole, which is reprotoxic in female rats

Predicted pesticide	Model	Evaluation	Detected structural features	Selected relevant analog structures	
*Epoxiconazole* CAS no. 135319‐73‐2 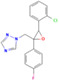	RFR v2	TP	Benzene, 1‐halo‐, 4‐oxymethyl‐ 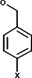	*Oxiconazole* *Positive* for RFR 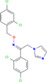	*Econazole* *Negative* for RFR 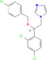
	RMR v2	FP	Benzene, 1‐fluoro‐ 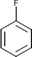	*Terconazole* *Positive* for RMR 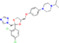	*Econazole* *Negative* for RMR For structure, see above
			Benzene, 1‐alkyl‐,2‐halo‐ 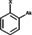		
			Benzene, 1‐alkyl‐,2‐chloro‐ 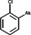		
			Benzene, 1‐alkyl‐,4‐halo‐ 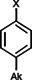		

Hence, the low reliability of the model predictions suggests that an expert review is necessary in predicting reprotoxic conazole fungicides within a chemical class that is mainly negative for toxic effects on reproduction.

#### 3.4.3 Summary

The conazole case study illustrates quite well the general problems of the QSAR prediction for pesticides using the Leadscope software. One of the main issues is that the identified structural features only cover part of the pesticide molecule. In the case of the conazoles, mainly benzene structures were identified. Therefore, an expert review is recommended, especially in the case of poor structural coverage. Relevant analog structures (inside and outside the Leadscope database) should also be taken into account. Additionally, it is important for the assessment to confirm that the probability score is not directly related to the reliability of the prediction. Overall, a majority of the pesticides fell outside the AD of the model. This is due to the fact that for many pesticides not all property descriptors, not at least one structural feature and/or not at least one analogous substance could be identified in the training set.

### 3.5 CASE ultra

In the following, the predictions of four selected CASE Ultra models (see Section 2.2.5) are examined. All models are statistically based structural alert models that use different data sets based on the respective endpoint. The classification is based on the alerts from which the probability is calculated. If an alert is assigned to the pesticide, the prediction can be positive or inconclusive. If there is no alert, the prediction is negative or out of domain. A known positive or known negative prediction can occur in both cases.

#### 3.5.1 Evaluation

The proportion of pesticides for which no prediction could be made (UNKNOWN), because they were either outside the AD of the model (out of domain) or the data were inconclusive (inclusive), was between 29 and 67% depending on the model (see Table 16 and Table S14). As a result, between 17 and 67% of reprotoxic pesticides were not recognized (see Table S14).

**TABLE 16:** The results of evaluation of the four tested CASE Ultra models FDYSM_Rabbit, FDYSM_Rat, FFERT_Rat and MFERT_Rat via typical parameters

Model	# FN	# FP	# TN	# TP	# UNKNOWN	SEN	SPC	BA	ACC
FDYSM_RABBIT	4	18	75	5	208	0.56	0.81	0.68	0.78
FDYSM_RAT	19	75	115	10	91	0.34	0.61	0.48	0.57
FFERT_RAT	2	18	132	1	157	0.33	0.88	0.61	0.87
MFERT_RAT	2	62	106	3	137	0.60	0.63	0.62	0.63

Abbreviations: ACC, accuracy; BA, balanced accuracy; FN, false negative; FP, false positive; SEN, sensitivity; SPC, specificity; TN, true negative; TP, true positive.

With the CASE Ultra models, there is also the case that tested pesticides also appear in the training data set of the respective model. This is then referred to as known positive/negative in the prediction. With the FDYSM_Rat and the MFRET_Rat model, 10 or 5 of these pesticides are nevertheless incorrectly predicted, which suggests a different data basis or interpretation of the data (see Table S15). Since the CASE Ultra models cannot access the underlying data, no further investigations were carried out.

All four CASE Ultra models showed a lower sensitivity (between 0.33 and 0.6) than specificity (between 0.61 and 0.87). The FDYSM_Rat model was particularly noticeable due to its high number of false negatives (19), 6 of which belonged to the triazoles. Since there is no external validation available for the CASE Ultra models, no comparison was possible.

The reprotoxicity is determined in the CASE Ultra models using the “Probability” value. The larger the value, the more reliable a positive prediction should theoretically be. However, this is not the case in any of the models, as can be seen in Figure S4.

#### 3.5.2 Alerts

The determination of the reprotoxicity of the CASE Ultra models is based on statistical structural alerts. These differ between the models. If no alert fits, the prediction is limited to known positive/negative, negative and out of domain. Otherwise, all predictions are possible, including a negative one. Several alerts are possible for each pesticide, but overall, no alert was assigned to over 60% of the pesticides for all models (see Table S16). Figure S5 shows the distribution of FN, FP, TN TP, and UNKNOWN per alert and model. The problem with the alerts used is that they are often very general and only cover very small sections of the molecule. Several alerts would always be required to cover the entire molecule, which is rarely the case. From the plot just described, therefore, it was not possible to select any alerts that would provide reliable predictions.

#### 3.5.3 Example

The prediction of reprotoxic potential of the triazoles by the FDYSM_Rat model should be used in the following to show the problems of the CASE Ultra models. The pesticide DB contains 21 triazoles of which 10 showed fetal dysmorphogenesis in rat studies relevant for ECHA classification. Three of them were predicted correctly, but six as negative and one was outside the AD of the model (see Table 17). Interestingly, the alert for all TPs was: C3H2‐C3‐c:cH:cH:c:cH (Alert ID 105), which describes an aromatic structure with at least one undefined substituent and a defined secondary substituent, which is a quaternary carbon followed by a secondary carbon. This alert only describes a small part of the molecule which is probably not very relevant for the toxicity mechanism as three non‐reprotoxic triazoles had the same alert (difenoconazole, flutriafol, myclobutanil). No alert could be assigned for the 6 FN triazoles, which indicates that there is a data gap here. Each prediction includes the 3 closest neighbors of the test chemical in the training set. In the case of the triazoles, there are some triazoles and imidazoles among these, but a similarity above 0.7 is never reached. Thus, these cannot be regarded as analog and therefore only have a limited significance.

**TABLE 17:** All triazoles of the pesticide DB that showed fetal dysmorphogenesis in ECHA classification‐relevant studies in rats and their prediction by the FDYSM_Rat model from CASE Ultra

Name	CAS no.	Structure	Prediction/probability/alert
Ipconazole	125225‐28‐7	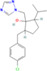	Negative/30.3/no alert
Metconazole	125116‐23‐6	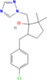	Negative/30.3/no alert
Paclobutrazol	76738‐62‐0	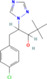	Negative/30.3/no alert
Penconazole	66246‐88‐6	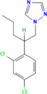	Negative/30.3/no alert
Tebuconazole	107534‐96‐3	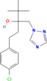	Negative/30.3/no alert
Triadimenol	55219‐65‐3	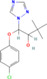	Negative/30.3/no alert
Epoxiconazole	133855‐98‐8	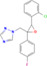	Out of domain/30.3/no alert
Bromuconazole	116255‐48‐2	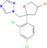	Positive/56/alert ID 105: C3H2‐C3‐c:cH:cH:c:cH 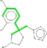
Cyproconazole	94361‐06‐5	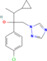	Positive/56/alert ID 105: C3H2‐C3‐c:cH:cH:c:cH 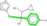
Propiconazole	60207‐90‐1	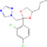	Positive/56/alert ID 105: C3H2‐C3‐c:cH:cH:c:cH 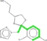

*Note*: When an alert was found, the relevant structure in the molecular pesticide structure is highlighted in green.

#### 3.5.4 Summary

The example shows the problem of the alerts within the CASE Ultra models. These form the basis of the prediction, but often only depict a small part of the molecular structure of the pesticides. This creates a high number of FPs. On the other hand, the critical structures are sometimes not recorded, or there is no suitable alert at all for reprotoxic pesticides, although all fragments are present in the data set. When evaluating the prediction, the alerts and their relevance should always be considered. The probability increases with an increasing number of alerts (not continuously) but is otherwise not a reliable indicator for the correctness of the prediction. When evaluating, the similarity of the 3 closest neighbors should also be considered. If this is more than 0.7, the substances can be considered analogous according to the model description. Overall, the assessment of the predictions of the CASE Ultra models also requires critical questioning by reprotoxicology experts.

### 3.6 Comparison

**FIGURE 5:**
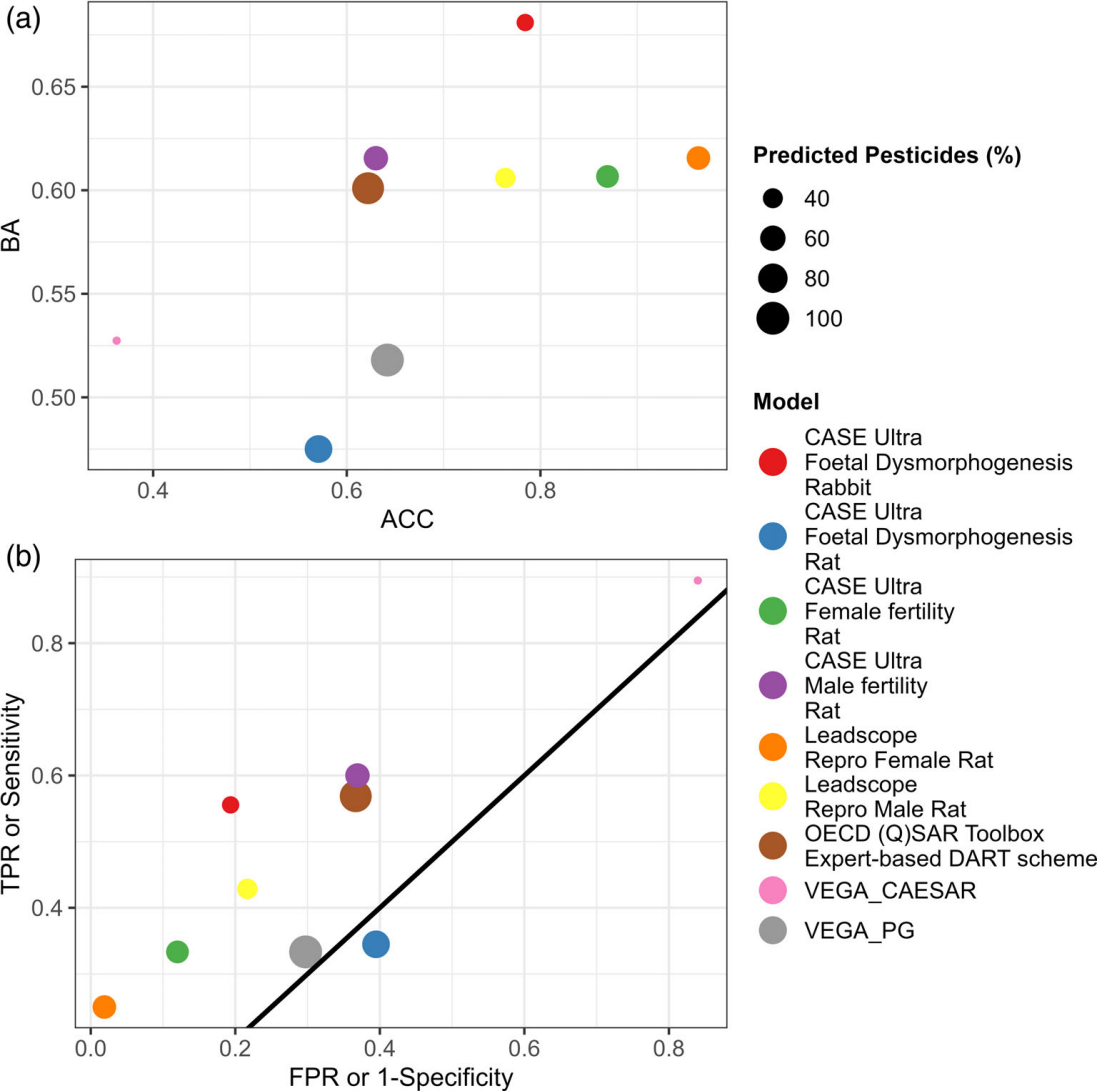
Plot of the accuracy against the balanced accuracy (a) and the FPR against the TPR (b) per model. The size of the points depends on the percentage of pesticides predicted. The black line shows the diagonal of the plot (TPR = FPR). The closer the points are to the diagonal, the more the model's prediction resembles a random process

There are several ways to compare the predictive power of the different models. The accuracy used for this is usually the one that should not be considered on its own in the case of an unbalanced training or test data set. This can be seen, for example, on the RFR model from Leadscope, which was rated the highest with an accuracy of 0.96. However, the sensitivity was only 0.25, which means that three quarters of all reprotoxic pesticides were not detected (see Figure 5 and Table S17). The balanced accuracy, which is the mean value of sensitivity and specificity, offers a better reference point. For the evaluation of the models, above all, sensitivity and specificity are decisive. Since the safety aspect plays a decisive role in predicting reprotoxicity and a low specificity is more tolerable than a low sensitivity, the focus is more on sensitivity. A typical representation for this is the ROC diagram in which the false positive rate (FPR, 1‐sensitivity) is plotted against the true positive rate (TPR, sensitivity). The closer the models are to the diagonal (black line), the more the prediction resembles a random process (see Figure 5b). Another important point to consider when assessing predictive power is how many of the pesticides were within the AD of the model and given a reliable score. In the models tested, this was between 100 and 22%.

The high number of “UNKNOWN” shows clearly that the majority of the models are not suitable for predicting pesticides, as these are outside the chemical space of the models. A sensitivity above 0.55 is only achieved with four models, whereas the CAESAR model has a specificity of only 0.16. The other three models (OQTB, CU_FDYS_Rabbit and CU_MFERT_Rat) achieve a specificity of at least 0.63. According to this statistical evaluation, all models are insufficient for predicting reprotoxicity or the partial aspects.

Also, in the overall comparison of the PG model with the DART scheme of the OECD (Q)SAR Toolbox, it becomes clear that the predictions of the models differ significantly, although both are originally based on the same decision tree. A detailed discussion of all differences can be found in Section 3.3.1. The Leadscope and CASE Ultra reprotoxicity models are based on the same database, but their models differ greatly (statistical QSAR vs. structural alert system), which also leads to large differences in the predictions. Both models predict toxicity of individual endpoints rather than overall reprotoxicity. However, this does not lead to an improvement in prediction reliability, as originally expected.

**FIGURE 6:**
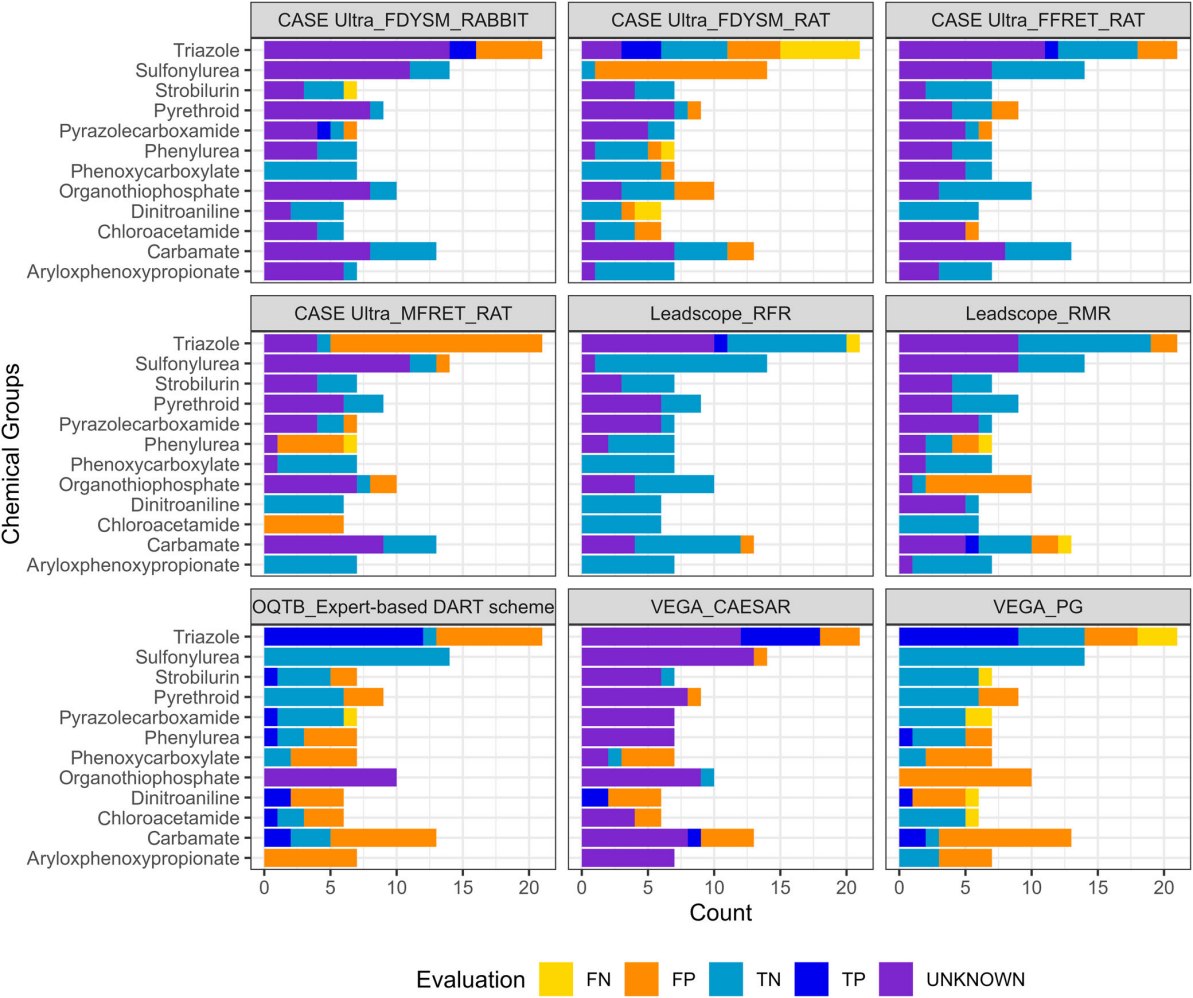
All bar plots show the distribution of FN, FP, TN, TP, and UNKNOWN per chemical group for a different prediction model. A more detailed description of the chemical groups can be found in Table 2

To find out whether the prediction quality differs between the chemical groups within the pesticide database, this was examined for the 12 largest chemical groups (see Table 2). Figure 6 shows the distribution of FP, FN, TN, TP, and UNKNOWN per chemical group for each model. These differed greatly between the models.

Of the 13 carbamates, 2 are classified as reprotoxic by the ECHA. Benfuracarb due to male reprotoxicity in the rat and carbendazim also due to male reprotoxicity in the rat but also fetal dysmorphogenesis in the rat and rabbit. When comparing the predictions of the PG model and the DART model of the OQTB for all carbamates, it is noticeable that the two reprotoxic pesticides were recognized as such, but most of the others were predicted false positives. In the PG model, almost all carbamates were also present in the training data set, which on the one hand suggests a different interpretation of the experimental data and on the other hand a general tendency of both models to classify carbamates as reprotoxic. The developmental toxicity shall be predicted by the CAESAR model. Most of the carbamates (eight pieces) were outside the model's AD and all others were predicted to be developmentally toxic, with only one actually being developmentally toxic. This phenomenon is not specific to carbamates, but in general the majority of pesticides was predicted by the CAESAR model to be developmentally toxic. In all CASE Ultra models, which each predict different aspects of reprotoxicity, more than half of the carbamates were not within AD or the prediction was inconclusive, including the reprotoxic pesticides. Only the FDYSM_Rat model predicted two carbamates as FP. In the Leadscope models, there were four and five carbamates outside the AD of the models. In the RFR model, the majority of carbamates TN and only one FP was predicted. Two reprotoxic ones were expected in the RMR model, of which carbendazim was recognized, but benfuracarb was predicted to be FN. Two carbamates were predicted in FP and four in TN.

Overall, it is noticeable that the prediction quality of the models, except for the CAESAR model, which generally tends to predict FP, differs between the chemical groups. If one compares the prediction quality of the chemical groups between the models, there are also major differences (e.g., dinitroaniline) and some models are then better suited than others for predicting the reprotoxicity of certain chemical groups.

By looking at the prediction quality in relation to selected groups, it becomes clear that the individual models can provide good predictions under certain conditions. When predicting the reprotoxicity using *in silico* models, it is therefore important to consider the predictions of several models and to weight them using the additional information provided in the report (alerts, similar compounds from the training data set) in order to arrive at a well‐founded opinion. The relevant additional information that should be analyzed is summarized in Table 18.

**TABLE 18:** Possible additional information on the prediction, which is made available in the reports

Information about…	Important questions
Structural alert/feature/predicted category	Does the selected structural fragment match the key functional groups of the pesticide?
Analog structures/similar compounds from training set	How similar are these compounds?
Data sources	Which source is the classification based on? Which effects are described in this source?

## 4 CONCLUSION

The aim of this paper was to test the performance of known models for predicting reproductive toxicity of pesticides and to use the results to analyze the strengths and weaknesses of the models. This resulted in suggested solutions for improving the models. The paper is intended to address three different target groups: *In silico* experts are to be made aware of the special problems of reprotoxicity, regulatory toxicologists are to be made aware of the limitations of the individual models and reprotoxicologists are to be made familiar with the *in silico* topic in order to point out what contribution they can still make.

The models used differed in several aspects (see Table 1 for details):

- Type of model (statistical model, expert rule‐based model or mixture)
- Training data set
- Endpoint (general reprotoxicity vs. selected reprotoxicity endpoints)

However, the comparison of the models does not allow any statement to be made as to which model type, training data set or endpoint is most suitable, since all models have major weaknesses in assessing reprotoxicity of pesticides. In four of the nine models, no reliable prediction can be made for over 50% of the pesticides and in five out of nine models, not even half of the reprotoxic pesticides are recognized (SEN < 0.5). In contrast, all models except the CAESAR model recognize at least 60% of the negative pesticides. Of course, the performance of the models differs but overall, no model is convincing if all three factors (number of predicted pesticides, SEN, SPC) are taken into account.

There are three main reasons for the poor performance of the models in relation to the pesticide database:

1. Many pesticides are not part of the chemical space of the models. For example, the CAESAR model, which is based on a drug database, cannot provide a reliable prediction for more than three quarters of all pesticides. Due to its database, it is only suitable to a limited extent for predicting pesticides. In general, however, this problem is due to a too small database with high‐quality reprotoxicity studies of pesticides. Therefore, larger databases based on uniform study designs would be needed to improve the models.
2. Definitions of reproductive toxicity vary. The unification of the assessment of toxicity is still a current issue for the *in vivo* area since the interpretations are also partly different here. For *in silico* toxicology, an important step here would no longer be to predict the entire reprotoxicity, but rather more easily definable, specific endpoints or effects. Even if this could not be shown with the models used, a better predictive power can be expected from endpoint‐specific models, since they are based on a smaller number of possible AOPs.
3. The partially insufficient definition of similarity within models. With the models provided by VEGA, the most similar molecules from the respective training data set are displayed in the report and used to calculate the reliability score (ADI). It is important to note that this analysis is independent of the prediction model. VEGA tended to overestimate the similarity of the structures (see Section 3.1.2 example). With the PG model and the DART scheme of the OECD (Q)SAR Toolbox, there were sometimes incorrect classifications into categories (see Sections 3.2.2 and 3.3.2). The Leadscope models include structural features, and the CASE Ultra models are alarm based. In both cases it would be desirable for the structural features or alerts to cover the entire molecular structure, but this is practically never achieved, which is more serious in the case of the CASE Ultra model. Since all prediction models, regardless of type, are essentially based on similarity, optimizing the calculation of similarity is a crucial step in improving the models. In order to describe similarity, there are more possibilities apart from fingerprints and descriptors, which should be used: AOPs, metabolism, receptor binding etc. At the structural level, the use of SMARTs or higher order substructures, that could even include metabolism information, would also be a possibility. It is crucial that the structures and properties relevant to the toxicity can be fully described using the selected parameters.

Despite all their weaknesses, the models can be of great use when used critically and the results compared to other models. Ensemble/consensus‐type approaches are suitable for this, which potentially make it possible to compensate for the weaknesses of one model with another. All models provide the reasons for the prediction (alerts) and/or similar molecules from the training data set in their respective report. This information usually allows a good assessment of the plausibility of the prediction, provides clues for further research and should therefore always be analyzed carefully. The DART scheme of the OECD (Q)SAR Toolbox and the PG model occupy a special position within the tested models, since they are both based on the expert‐known‐based decision tree by Wu et al. (2013). This gives a good overview of chemical groups with known reprotoxicity and can serve as a starting point for the development/inclusion of MOAs and AOPs.

All the points mentioned are of course suitable for improving prediction models, regardless of the type of toxicity. For reprotox, however, the conditions are more difficult overall due to the small amount of available and high‐quality data, the complexity of the underlying studies, the knowledge gaps regarding the modes of action and the point that reprotoxicity is a mixture of effects, which encompass a number of endpoints. Solving the problems just described is essential for the development of successful reprotoxicity models. Until then, using the models already available requires a critical look at the results based on reprotox expertise.

## Supporting information


**Data S1**. Supporting information.

**Data S2**. Supporting information.

## Data Availability

The data that supports the findings of this study are available in the supplementary material of this article.

## References

[bdr22062-bib-0001] Arena, V. C. , Sussman, N. B. , Mazumdar, S. , Yu, S. , & Macina, O. T. (2004). The utility of structure‐activity relationship (SAR) models for prediction and covariate selection in developmental toxicity: Comparative analysis of logistic regression and decision tree models. SAR and QSAR in Environmental Research, 15(1), 1–18. 10.1080/1062936032000169633 15113065

[bdr22062-bib-0002] Avram, M. J. (2020). Pharmacokinetic studies in pregnancy. Seminars in Perinatology, 44(3), 151227. 10.1016/j.semperi.2020.151227 32093881 PMC7323629

[bdr22062-bib-0003] Bantle, J. A. , Fort, D. J. , & James, B. L. (1989). Identification of developmental toxicants using the frog embryo Teratogenesis assay‐Xenopus (FETAX). Hydrobiologia, 188(1), 577–585. 10.1007/BF00027826

[bdr22062-bib-0004] Benfenati, E. (2020). Developmental/Reproductive Toxicity Library (PG) (version 1.1.0). QMRF.

[bdr22062-bib-0005] Berthold, M. R. , Cebron, N. , Dill, F. , Gabriel, T. R. , Kötter, T. , Meinl, T. , … Wiswedel, B. (2008). KNIME: The Konstanz information miner. In I. C. Preisach , H. Burkhardt , L. Schmidt‐Thieme , & R. Decker (Eds.), Data analysis, machine learning and applications Berlin. Heidelberg: KNIME: The Konstanz Information Miner.

[bdr22062-bib-0006] Cassano, A. , & Benfenati, E. (2010). Developmental toxicity model (CAESAR) – v. 2.1.7. QMRF.

[bdr22062-bib-0007] Cassano, A. , Manganaro, A. , Martin, T. , Young, D. , Piclin, N. , Pintore, M. , … Benfenati, E. (2010). CAESAR models for developmental toxicity. Chemistry Central Journal, 4 (Suppl 1), S4. 10.1186/1752-153X-4-S1-S4 20678183 PMC2913331

[bdr22062-bib-0008] Cereto‐Massagué, A. , Ojeda, M. J. , Valls, C. , Mulero, M. , Garcia‐Vallvé, S. , & Pujadas, G. (2015). Molecular fingerprint similarity search in virtual screening. Methods, 71, 58–63. 10.1016/j.ymeth.2014.08.005 25132639

[bdr22062-bib-0009] Chakravarti, S. K. , Saiakhov, R. D. , & Klopman, G. (2012). Optimizing predictive performance of CASE ultra expert system models using the applicability domains of individual toxicity alerts. Journal of Chemical Information and Modeling, 52(10), 2609–2618. 10.1021/ci300111r 22947043

[bdr22062-bib-0010] Cioffi, G. (2019a). RP_FDYSM_RABBIT, fetal Dysmorphogenesis, Rabbit, Model version, 1.7.0.5.257.500. QMRF.

[bdr22062-bib-0011] Cioffi, G. (2019b). RP_FDYSM_RAT, fetal Dysmorphogenesis, Rat, Model version, 1.7.0.5.892.450. QMRF.

[bdr22062-bib-0012] Cioffi, G. (2019c). RP_FFERT_RAT, male fertility, Rat, Model version, 1.7.0.5.226.550. QMRF.

[bdr22062-bib-0013] Cioffi, G. (2019d). RP_MFERT_RAT, male fertility, Rat, Model version, 1.7.0.5.360.500. QMRF.

[bdr22062-bib-0014] Cronin, M. T. D. , & Worth, A. P. (2008). (Q)SARs for predicting effects relating to reproductive toxicity. QSAR & Combinatorial Science, 27(1), 91–100. 10.1002/qsar.200710118

[bdr22062-bib-0015] Danishuddin, M. , & Khan, A. U. (2016). Descriptors and their selection methods in QSAR analysis: Paradigm for drug design. Drug Discovery Today, 21(8), 1291–1302. 10.1016/j.drudis.2016.06.013 27326911

[bdr22062-bib-0016] De Schaepdrijver, L. M. , Annaert, P. P. J. , & Chen, C. L. (2019). Ontogeny of ADME processes during postnatal development in man and preclinical species: A comprehensive review. Drug Metabolism and Disposition, 47(3), 295. 10.1124/dmd.118.084350 30770345

[bdr22062-bib-0017] Enoch, S. J. , Hasarova, Z. , Cronin, M. T. D. , Bridgwood, K. , Rao, S. , Kluxen, F. M. , & Frericks, M. (2022). Sub‐structure‐based category formation for the prioritisation of genotoxicity hazard assessment for pesticide residues: Sulphonyl ureas. Regulatory Toxicology and Pharmacology, 129, 105115. 10.1016/j.yrtph.2022.105115 35017022

[bdr22062-bib-0018] Floris, M. , Manganaro, A. , Nicolotti, O. , Medda, R. , Mangiatordi, G. F. , & Benfenati, E. (2014). A generalizable definition of chemical similarity for read‐across. Journal of Cheminformatics, 6(1), 39. 10.1186/s13321-014-0039-1 25383097 PMC4212147

[bdr22062-bib-0019] Hansch, C. , & Fujita, T. (1964). P‐σ‐π analysis. A method for the correlation of biological activity and chemical structure. Journal of the American Chemical Society, 86(8), 1616–1626. 10.1021/ja01062a035

[bdr22062-bib-0020] Hanser, T. , Barber, C. , Guesné, S. , Marchaland, J. F. , & Werner, S. (2019). Applicability domain: Towards a more formal framework to express the applicability of a model and the confidence in individual predictions. In H. Hong (Ed.), Advances in computational toxicology: Methodologies and applications in regulatory science (pp. 215–232). Cham: Springer International Publishing. 10.1007/978-3-030-16443-0_11

[bdr22062-bib-0021] Hewitt, M. , Ellison, C. M. , Enoch, S. J. , Madden, J. C. , & Cronin, M. T. D. (2010). Integrating (Q)SAR models, expert systems and read‐across approaches for the prediction of developmental toxicity. Reproductive Toxicology, 30(1), 147–160. 10.1016/j.reprotox.2009.12.003 20006701

[bdr22062-bib-0022] Khera, K. S. (1987). Maternal toxicity in humans and animals: Effects on fetal development and criteria for detection. Teratogenesis, Carcinogenesis, and Mutagenesis, 7(3), 287–295. 10.1002/tcm.1770070309 2888207

[bdr22062-bib-0023] King, C. T. , Rogers, P. D. , Cleary, J. D. , & Chapman, S. W. (1998). Antifungal therapy during pregnancy. Clinical Infectious Diseases, 27(5), 1151–1160. 10.1086/514977 9827262

[bdr22062-bib-0024] Kjærstad, M. B. , Taxvig, C. , Nellemann, C. , Vinggaard, A. M. , & Andersen, H. R. (2010). Endocrine disrupting effects in vitro of conazole antifungals used as pesticides and pharmaceuticals. Reproductive Toxicology, 30(4), 573–582. 10.1016/j.reprotox.2010.07.009 20708073

[bdr22062-bib-0025] Leadscope . (2021). B‐3. Reproductive Toxicity Suite. Leadscope Model Applier Documentation, *Version 3.1* .

[bdr22062-bib-0026] Lopez‐Rangel, E. , & Van Allen, M. I. (2005). Prenatal exposure to fluconazole: An identifiable dysmorphic phenotype. Birth Defects Research Part A: Clinical and Molecular Teratology, 73(11), 919–923. 10.1002/bdra.20189 16265639

[bdr22062-bib-0027] Matthews, E. J. , Kruhlak, N. L. , Cimino, M. C. , Benz, R. D. , & Contrera, J. F. (2006a). An analysis of genetic toxicity, reproductive and developmental toxicity, and carcinogenicity data: I. Identification of carcinogens using surrogate endpoints. Regulatory Toxicology and Pharmacology, 44(2), 83–96. 10.1016/j.yrtph.2005.11.003 16386343

[bdr22062-bib-0028] Matthews, E. J. , Kruhlak, N. L. , Cimino, M. C. , Benz, R. D. , & Contrera, J. F. (2006b). An analysis of genetic toxicity, reproductive and developmental toxicity, and carcinogenicity data: II. Identification of genotoxicants, reprotoxicants, and carcinogens using in silico methods. Regulatory Toxicology and Pharmacology, 44(2), 97–110. 10.1016/j.yrtph.2005.10.004 16352383

[bdr22062-bib-0029] Matthews, E. J. , Kruhlak, N. L. , Daniel Benz, R. , & Contrera, J. F. (2007). A comprehensive model for reproductive and developmental toxicity hazard identification: I. Development of a weight of evidence QSAR database. Regulatory Toxicology and Pharmacology, 47(2), 115–135. 10.1016/j.yrtph.2006.11.002 17207562

[bdr22062-bib-0030] Matthews, E. J. , Kruhlak, N. L. , Daniel Benz, R. , Ivanov, J. , Klopman, G. , & Contrera, J. F. (2007). A comprehensive model for reproductive and developmental toxicity hazard identification: II. Construction of QSAR models to predict activities of untested chemicals. Regulatory Toxicology and Pharmacology, 47(2), 136–155. 10.1016/j.yrtph.2006.10.001 17175082

[bdr22062-bib-0031] Mellor, C. L. , Marchese Robinson, R. L. , Benigni, R. , Ebbrell, D. , Enoch, S. J. , Firman, J. W. , … Cronin, M. T. D. (2019). Molecular fingerprint‐derived similarity measures for toxicological read‐across: Recommendations for optimal use. Regulatory Toxicology and Pharmacology, 101, 121–134. 10.1016/j.yrtph.2018.11.002 30468762

[bdr22062-bib-0032] Mogensen, D. M. , Pihl, M. B. , Skakkebæk, N. E. , Andersen, H. R. , Juul, A. , Kyhl, H. B. , … Jensen, T. K. (2017). Prenatal exposure to antifungal medication may change anogenital distance in male offspring: A preliminary study. Environmental Health, 16(1), 68. 10.1186/s12940-017-0263-z 28637461 PMC5480178

[bdr22062-bib-0033] Nitzsche, D. (2017). Effect of maternal feed restriction on prenatal development in rats and rabbits ‐ A review of published data. Regulatory Toxicology and Pharmacology, 90, 95–103. 10.1016/j.yrtph.2017.08.009 28822876

[bdr22062-bib-0034] OECD . (2001). Test no. 416: Two‐generation reproduction toxicity. OECD Publishing. 10.1787/9789264070868-en

[bdr22062-bib-0035] OECD . (2006). Report on the regulatory uses and applications in OECD member countries of (quantitative) structure‐activity relationship [(Q)SAR] models in the assessment of new and existing chemicals. OECD Papers, *vol*. 6/11. 10.1787/oecd_papers-v6-art37-en

[bdr22062-bib-0036] OECD . (2018a). *Test no. 414: Prenatal developmental toxicity study* (Vol.). OECD Publishing. 10.1787/9789264070820-en

[bdr22062-bib-0037] OECD . (2018b). Test no. 443: Extended one‐generation reproductive toxicity study. OECD Publishing. 10.1787/9789264185371-en

[bdr22062-bib-0038] OECD (Q)SAR Toolbox . (2020). Description of the DART scheme, v1.4. Procter & Gamble. Bourgas, Bulgaria: Laboratory of Mathematical Chemistry (LMC).

[bdr22062-bib-0039] Piersma, A. H. , Genschow, E. , Verhoef, A. , Spanjersberg, M. Q. , Brown, N. A. , Brady, M. , … Spielmann, H. (2004). Validation of the postimplantation rat whole‐embryo culture test in the international ECVAM validation study on three in vitro embryotoxicity tests. Alternatives to Laboratory Animals, 32(3), 275–307. 10.1177/026119290403200307 15588168

[bdr22062-bib-0040] R Core Team . (2019). R: A language and environment for statistical computing. In R Foundation for Statistical Computing. https://www.R-project.org/

[bdr22062-bib-0041] Raies, A. B. , & Bajic, V. B. (2016). In silico toxicology: Computational methods for the prediction of chemical toxicity. Wiley Interdisciplinary Reviews: Computational Molecular Science, 6(2), 147–172. 10.1002/wcms.1240 27066112 PMC4785608

[bdr22062-bib-0042] Russell, W. M. S. , & Burch, R. L. (1959). The principles of humane experimental technique. Methuen.

[bdr22062-bib-0043] Seiler, A. E. M. , & Spielmann, H. (2011). The validated embryonic stem cell test to predict embryotoxicity in vitro. Nature Protocols, 6(7), 961–978. 10.1038/nprot.2011.348 21720311

[bdr22062-bib-0044] Selderslaghs, I. W. , Van Rompay, A. R. , De Coen, W. , & Witters, H. E. (2009). Development of a screening assay to identify teratogenic and embryotoxic chemicals using the zebrafish embryo. Reproductive Toxicology, 28(3), 308–320. 10.1016/j.reprotox.2009.05.004 19447169

[bdr22062-bib-0045] Škuta, C. , Cortés‐Ciriano, I. , Dehaen, W. , Kříž, P. , van Westen, G. J. P. , Tetko, I. V. , … Svozil, D. (2020). QSAR‐derived affinity fingerprints (part 1): Fingerprint construction and modeling performance for similarity searching, bioactivity classification and scaffold hopping. Journal of Cheminformatics, 12(1), 39. 10.1186/s13321-020-00443-6 33431038 PMC7260783

[bdr22062-bib-0046] Society for the Advancement of Adverse Outcome Pathways . (2022). Adverse outcome pathways for Reprotox. https://aopwiki.org/

[bdr22062-bib-0047] Tasnif, Y. , Morado, J. , & Hebert, M. F. (2016). Pregnancy‐related pharmacokinetic changes. Clinical Pharmacology and Therapeutics, 100(1), 53–62. 10.1002/cpt.382 27082931

[bdr22062-bib-0048] Tetro, N. , Moushaev, S. , Rubinchik‐Stern, M. , & Eyal, S. (2018). The placental barrier: The gate and the fate in drug distribution. Pharmaceutical Research, 35(4), 71. 10.1007/s11095-017-2286-0 29476301

[bdr22062-bib-0049] Theunissen, P. T. , Beken, S. , Beyer, B. K. , Breslin, W. J. , Cappon, G. D. , Chen, C. L. , … Piersma, A. H. (2016). Comparison of rat and rabbit embryo‐fetal developmental toxicity data for 379 pharmaceuticals: On the nature and severity of developmental effects. Critical Reviews in Toxicology, 46(10), 900–910. 10.1080/10408444.2016.1224807 27848393 PMC8865449

[bdr22062-bib-0050] U.S. EPA . (2005a). 2,4‐D HED's revised human health risk assessment for the reregistration eligibility decision (RED) revised to reflect public comments.

[bdr22062-bib-0058] U.S. EPA. (2005b). 2,4‐D Reregistration Eligibility Decision. (EPA 738‐R‐05‐002). Washington, DC: U.S. EPA.

[bdr22062-bib-0052] Valerio, L. G. (2009). In silico toxicology for the pharmaceutical sciences. Toxicology and Applied Pharmacology, 241(3), 356–370. 10.1016/j.taap.2009.08.022 19716836

[bdr22062-bib-0053] Venkatapathy, R. , & Wang, N. C. Y. (2013). Developmental toxicity prediction. In B. Reisfeld & A. N. Mayeno (Eds.), Computational toxicology (Vol. II, pp. 305–340). Totowa, NJ: Humana Press. 10.1007/978-1-62703-059-5_14

[bdr22062-bib-0054] Wickham, H. (2016). ggplot2: Elegant graphics for data analysis. In Springer‐Verlag.

[bdr22062-bib-0055] Wu, S. , Fisher, J. , Naciff, J. , Laufersweiler, M. , Lester, C. , Daston, G. , & Blackburn, K. (2013). Framework for identifying chemicals with structural features associated with the potential to act as developmental or reproductive toxicants. Chemical Research in Toxicology, 26(12), 1840–1861. 10.1021/tx400226u 24206190

[bdr22062-bib-0056] Yang, H. , Lou, C. , Li, W. , Liu, G. , & Tang, Y. (2020). Computational approaches to identify structural alerts and their applications in environmental toxicology and drug discovery. Chemical Research in Toxicology, 33(6), 1312–1322. 10.1021/acs.chemrestox.0c00006 32091207

[bdr22062-bib-0057] Zarn, J. A. , Brüschweiler, B. J. , & Schlatter, J. R. (2003). Azole fungicides affect mammalian steroidogenesis by inhibiting sterol 14 alpha‐demethylase and aromatase. Environmental Health Perspectives, 111(3), 255–261. 10.1289/ehp.5785 12611652 PMC1241380

