# Supplementary material for: Review of the state of science and evaluation of currently available in silico prediction models for reproductive and developmental toxicity: A case study on pesticides
Source: Birth Defects Res. 2022 Jun 24;114(14):812–42. doi: 10.1002/bdr2.2062 (PMC9545887; doi:10.1002/bdr2.2062)
Supplement: Supplementary file 1 — Data S1. Supporting information. [file BDR2-114-812-s001.docx]

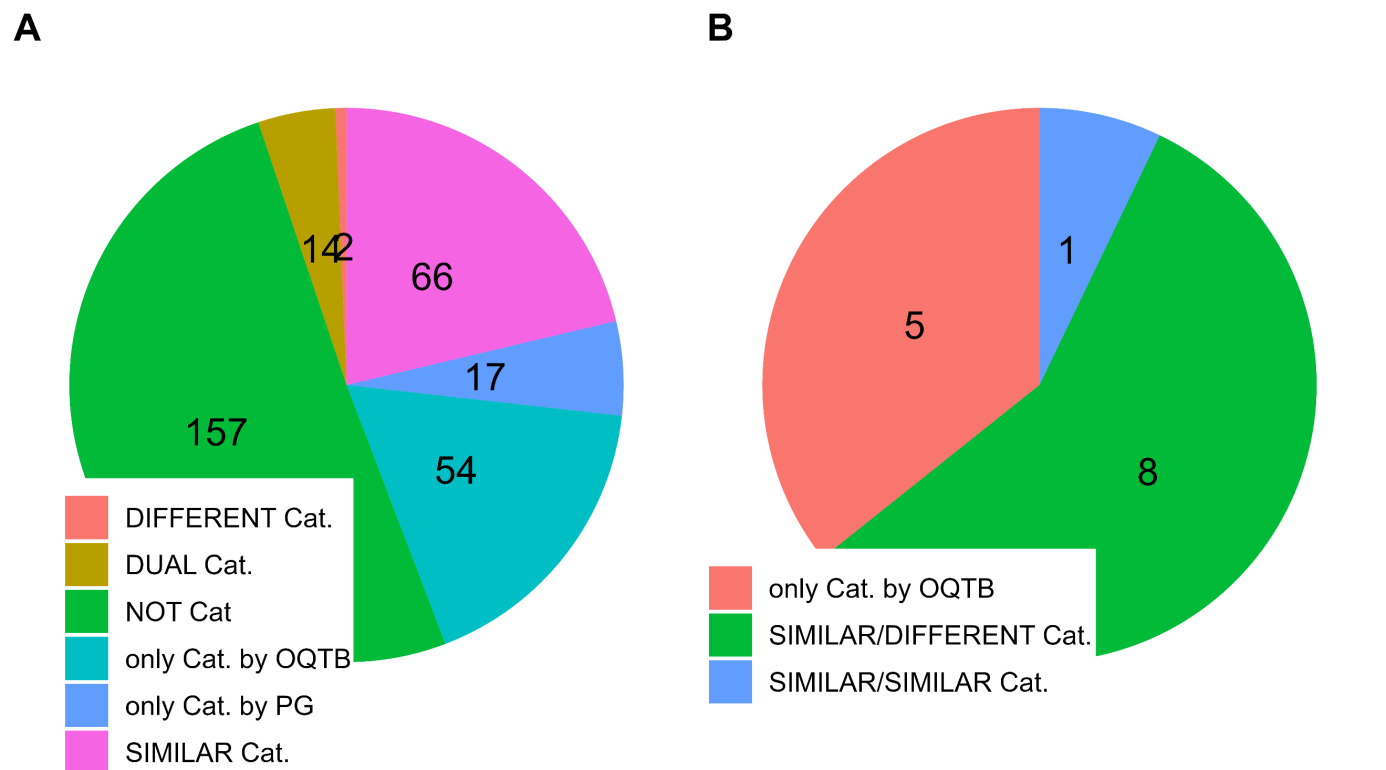


Figure S1: Comparison between the categorization of the PG model and the DART scheme of the OECD (Q)SAR Toolbox (OQTB). (A) Comparison of all 310 pesticides of the pesticide DB; (B) Comparison of the pesticides only categorised by the OQTB. Cat.:Categorized


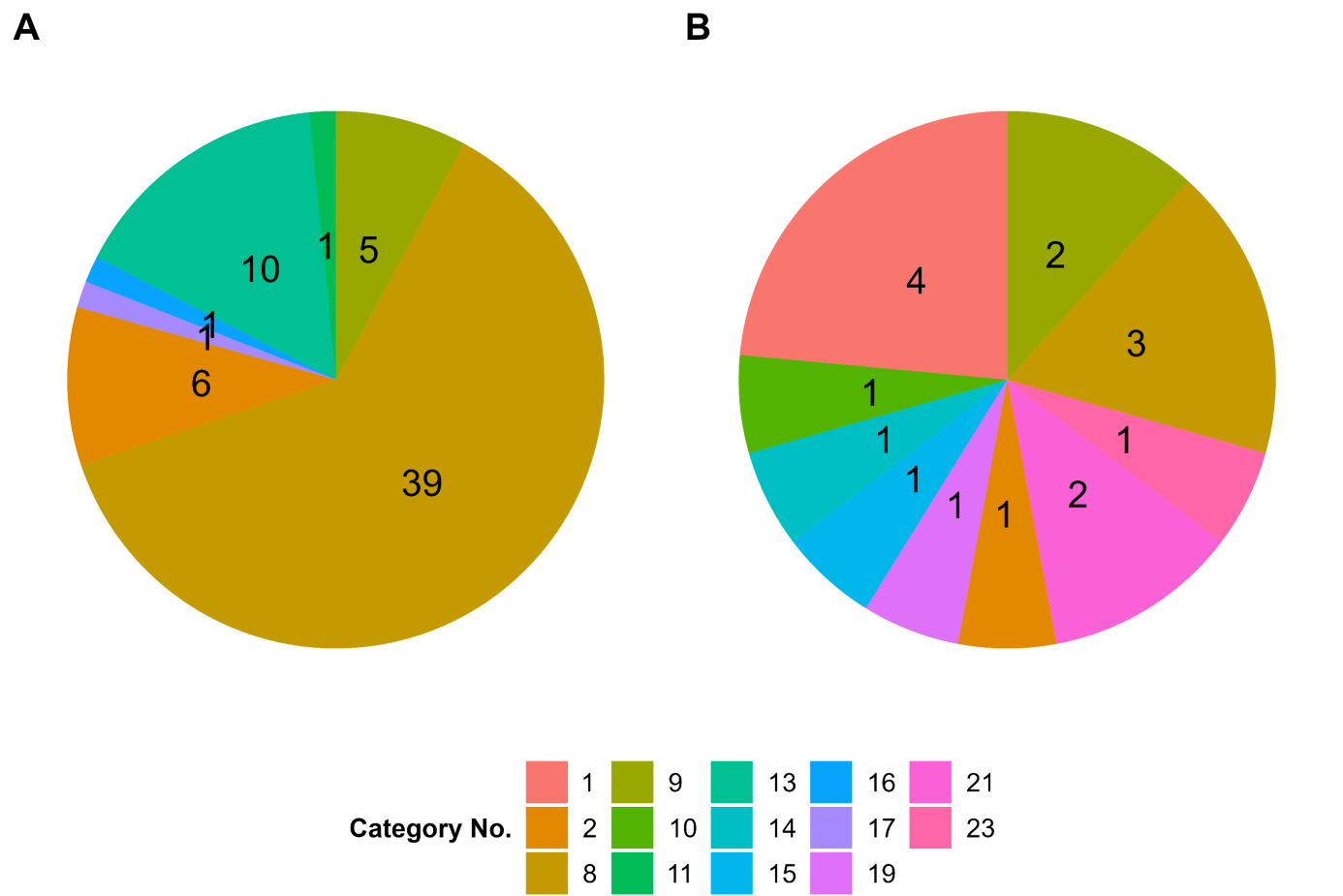


Figure S2: Comparison between the categorization of the PG model and the DART scheme of the OECD (Q)SAR Toolbox (OQTB) for the pesticides only categorized by one of the prediction models. (A) Distribution of categories of pesticides only categorized by OQTB; (B) Distribution of categories of pesticides only categorized by the PG model.


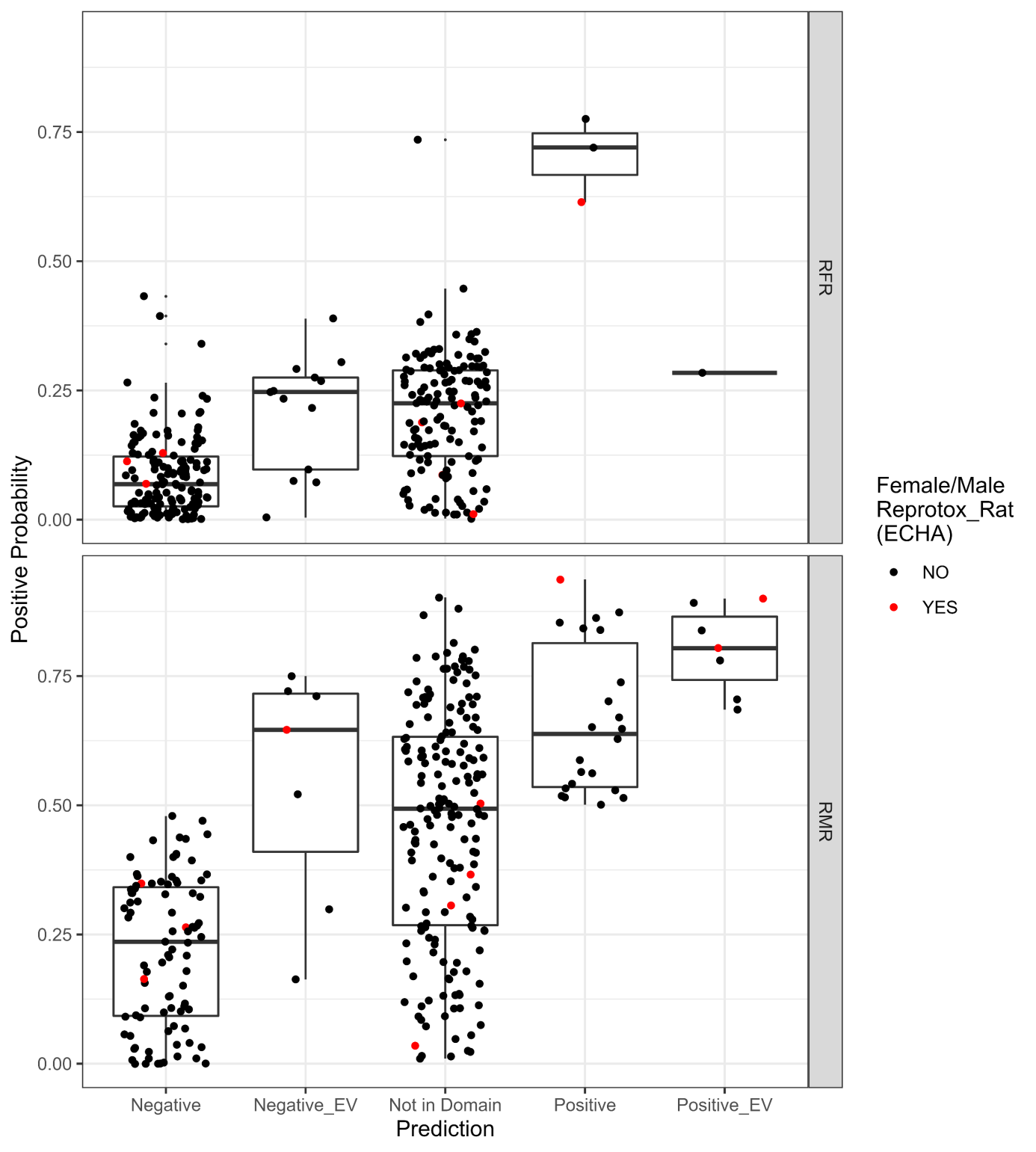


Figure S3: Distribution of the "Positive Probability" related to the different predictions in the Repro Female Rat (RFR) and Repro Male Rat (RMR) model from Leadscope. In addition to showing the distribution by boxplots, each pesticide is represented by a dot. The color of the dots refers to the endpoint specific toxicity derived from the studies used for the ECHA classification.


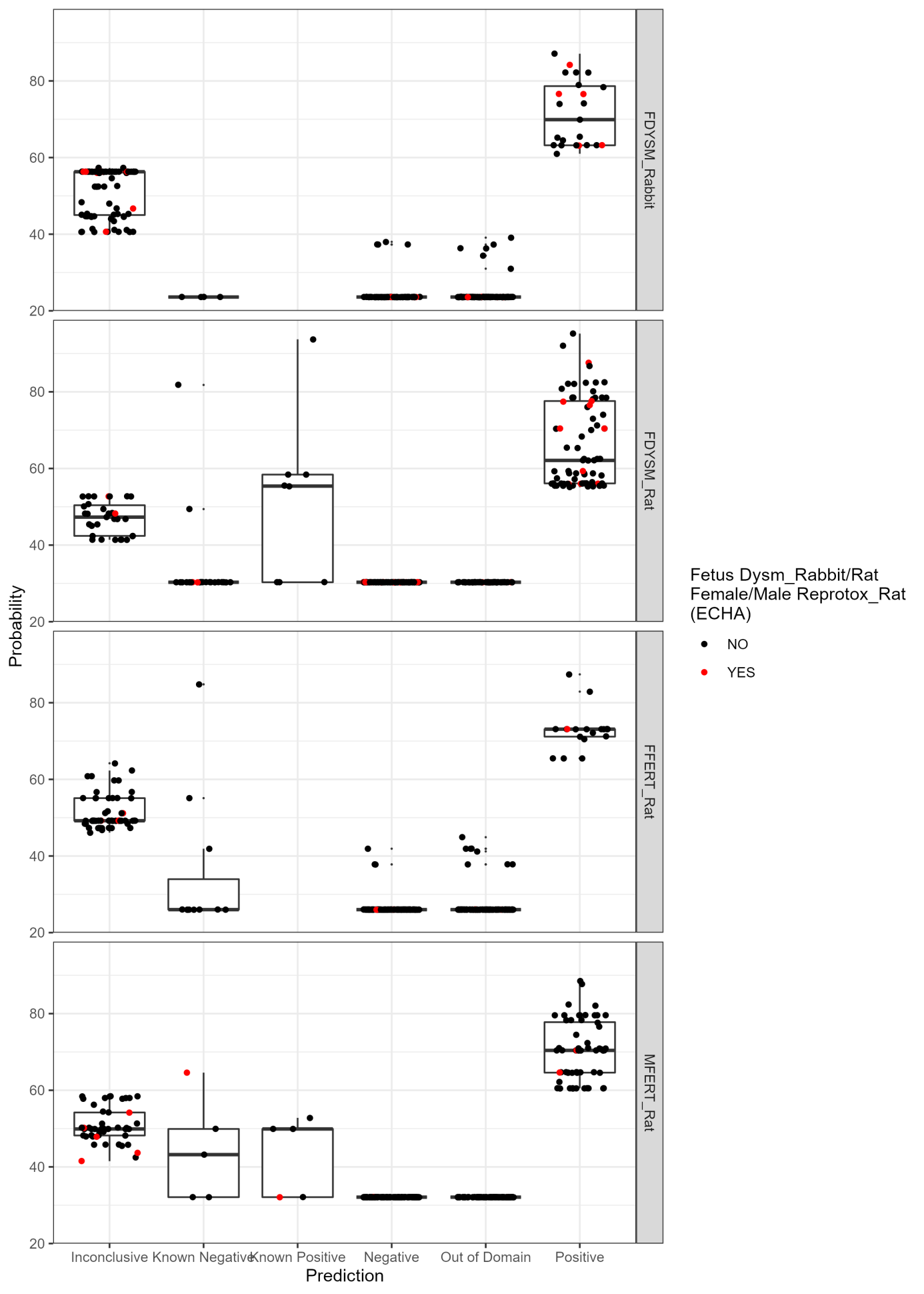


Figure S4: Distribution of the "Positive Probability" related to the different predictions of the four tested CASE Ultra models. In addition to showing the distribution by boxplots, each pesticide is represented by a dot. The color of the dots refers to the endpoint specific toxicity derived from the studies used for the ECHA classification.


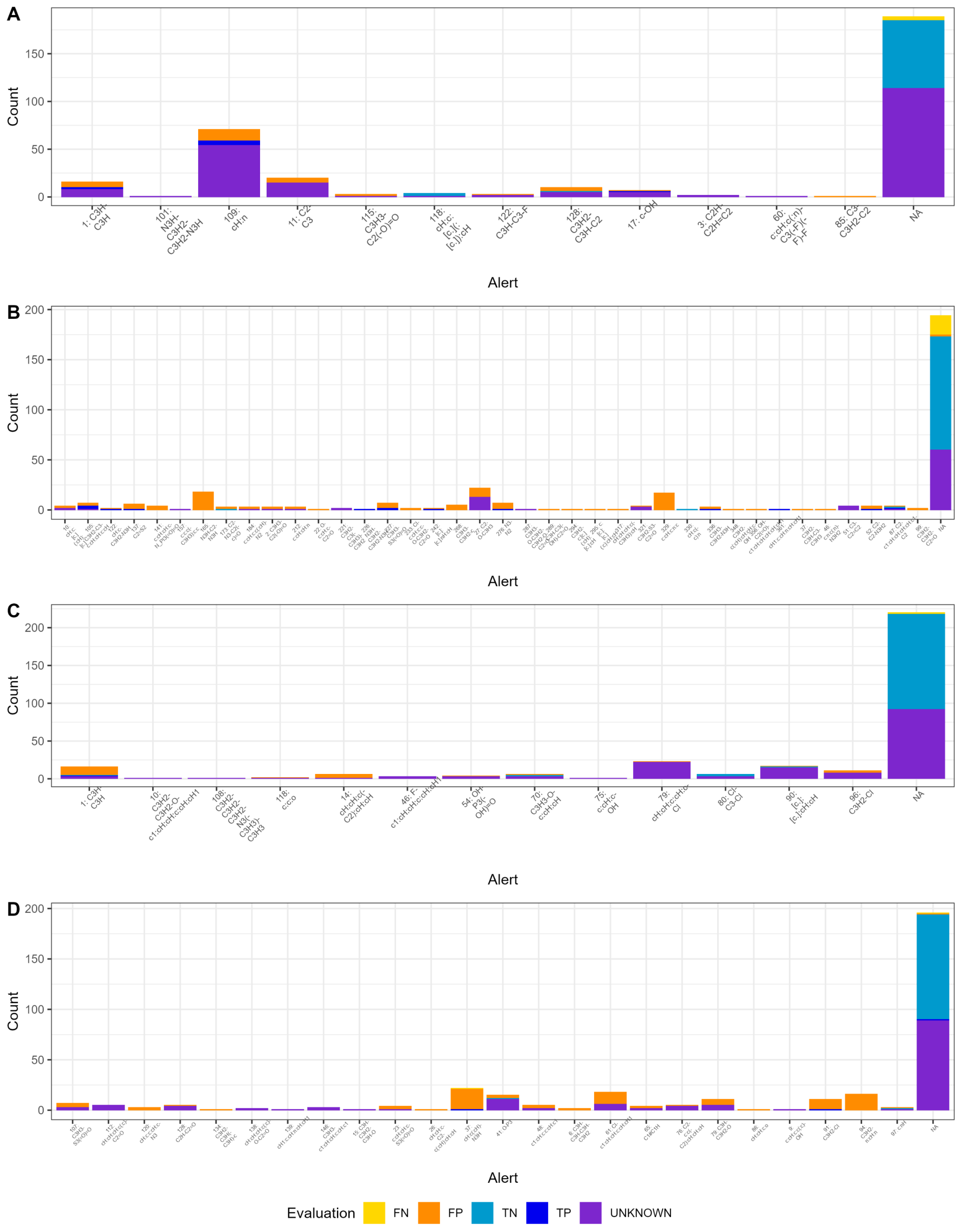


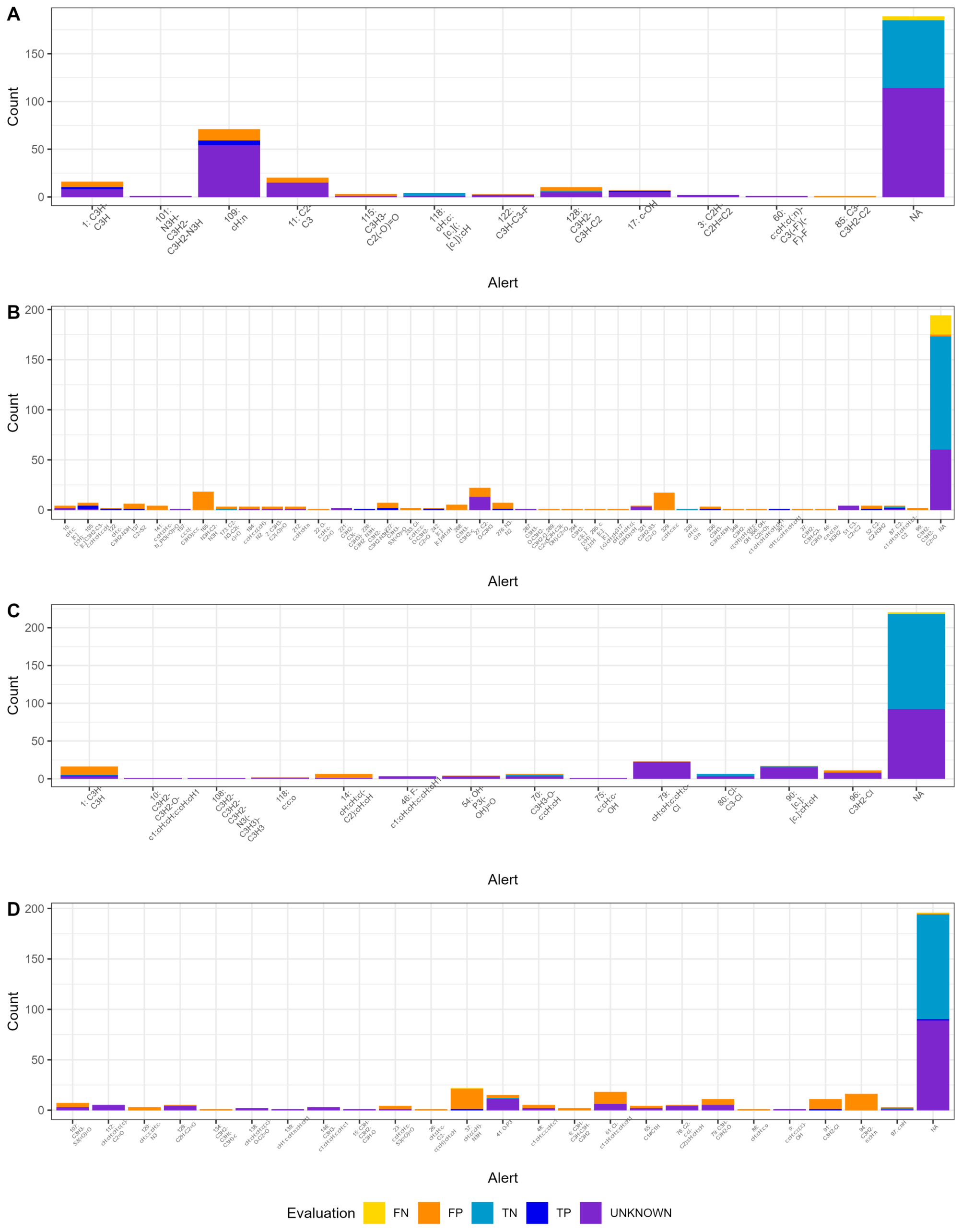


Figure S5: Evaluation of the predictions divided by the alerts for the four tested CASE Ultra models. FN: False negative; FP: False positive; TN: True negative; TP: True positive
